# Build Your Own Mushroom Soil: Microbiota Succession and Nutritional Accumulation in Semi-Synthetic Substratum Drive the Fructification of a Soil-Saprotrophic Morel

**DOI:** 10.3389/fmicb.2021.656656

**Published:** 2021-05-24

**Authors:** Hao Tan, Yang Yu, Jie Tang, Tianhai Liu, Renyun Miao, Zhongqian Huang, Francis M. Martin, Weihong Peng

**Affiliations:** ^1^National-Local Joint Engineering Laboratory of Breeding and Cultivation of Edible and Medicinal Fungi, Mushroom Research Center, Soil and Fertilizer Institute, Sichuan Academy of Agricultural Sciences, Chengdu, China; ^2^School of Life Sciences, Jiangnan University, Wuxi, China; ^3^Scientific Observing and Experimental Station of Agro-Microbial Resource and Utilization in Southwest China, Ministry of Agriculture and Rural Affairs, Chengdu, China; ^4^Université de Lorraine, INRAE, UMR Interactions Arbres/Microorganismes, Centre INRAE Grand Est-Nancy, Champenoux, France; ^5^Beijing Advanced Innovation Center for Tree Breeding by Molecular Design, Beijing Forestry University, Beijing, China

**Keywords:** soil-saprotrophic mushroom, semi-synthetic substratum, inoculated microbiota, lipid, nitrogen

## Abstract

Black morel, a widely prized culinary delicacy, was once an uncultivable soil-saprotrophic ascomycete mushroom that can now be cultivated routinely in farmland soils. It acquires carbon nutrients from an aboveground nutritional supplementation, while it remains unknown how the morel mycelium together with associated microbiota in the substratum metabolizes and accumulates specific nutrients to support the fructification. In this study, a semi-synthetic substratum of quartz particles mixed with compost was used as a replacement and mimic of the soil. Two types of composts (C1 and C2) were used, respectively, plus a bare-quartz substratum (NC) as a blank reference. Microbiota succession, substrate transformation as well as the activity level of key enzymes were compared between the three types of substrata that produced quite divergent yields of morel fruiting bodies. The C1 substratum, with the highest yield, possessed higher abundances of *Actinobacteria* and *Chloroflexi*. In comparison with C2 and NC, the microbiota in C1 could limit over-expansion of microorganisms harboring N-fixing genes, such as *Cyanobacteria*, during the fructification period. Driven by the microbiota, the C1 substratum had advantages in accumulating lipids to supply morel fructification and maintaining appropriate forms of nitrogenous substances. Our findings contribute to an increasingly detailed portrait of microbial ecological mechanisms triggering morel fructification.

## Introduction

**FIGURE 1:**
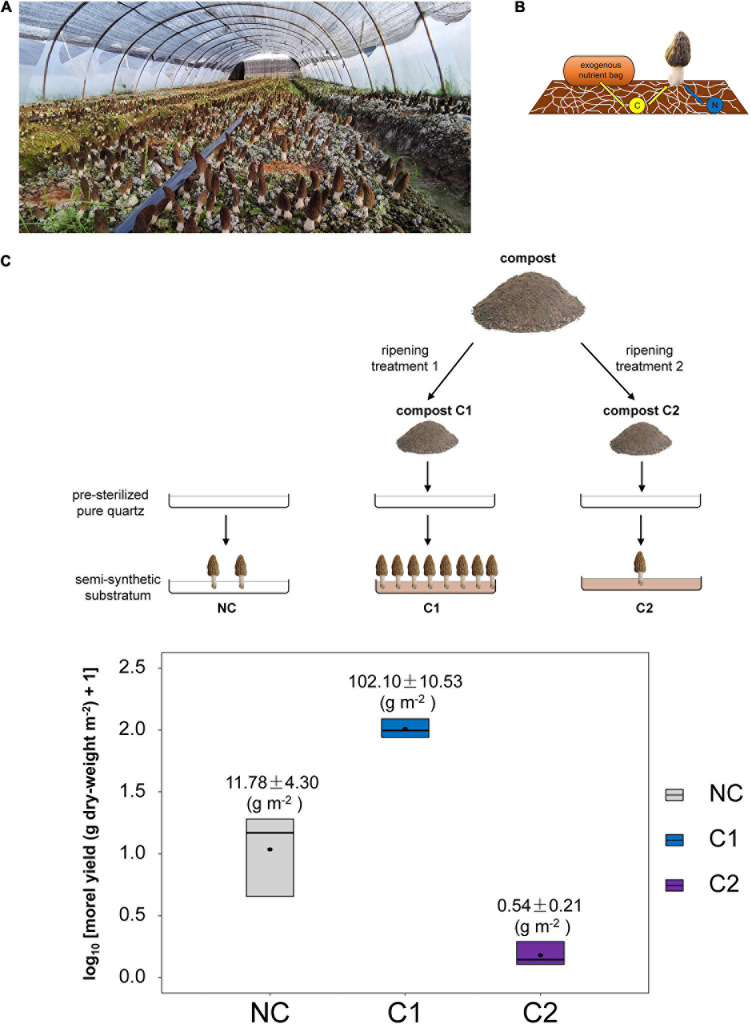
**(A)** Soil substratum, the so-called mushroom-bed for the fructification of black morel in large-scale agricultural production. **(B)** The soil C source to support morel fructification was acquired initially from the aboveground nutritional supplementation of exogenous nutrient bags, while essential N nutrients were mostly from the soil itself, as reported previously (Tan et al., 2019). The exhausted exogenous nutrient bags could be either removed before fructification or left on the soil surface until the finish of fruiting-body harvest. The schematic diagram was modified from the cover-illustration of issue 10, volume 21 of *Environmental Microbiology* (2019). **(C)** Schematic diagram of the experimental design in this study and the morel yield obtained from the three types of semi-synthetic substrata.

Species in the ascomycete genus *Morchella* are culinary delicacies widely prized in the world, commonly known as morels (Pilz et al., 2007). With great ecological and economic importance, the nutritional strategies of morels vary from soil-saprotrophic to biotrophic (Larson et al., 2016; Loizides, 2017; Longley et al., 2019). Their production was once quite dependent on collection from wild forests (Pilz et al., 2004, 2007). *Morchella importuna* (Kuo, O’Donnell, and Volk) is a black morel species believed to be soil-saprotrophic while able to form a facultative mycorrhizal-like association with plant-roots (Dahlstrom et al., 2000; Loizides, 2017). It was recently domesticated as a commercial mushroom-crop (Peng et al., 2016) with a rapidly growing scale of cultivation all over the world (Liu et al., 2018; Sambyal and Singh, 2021). With a unique form of nutritional supplementation aboveground composed of wheat grains and rice husks (Tan et al., 2019), *M. importuna* can produce ascocarps (fruiting bodies) with high and stable yield from ordinary farmland soils, the so-called mushroom-bed substratum (Figure 1A; Liu et al., 2018). Previous studies revealed that organic C was transferred from the aboveground nutritional supplementation toward the soil substratum, while the N nutrients for morel seemed to be obtained from the soil substratum itself (Figure 1B; Tan et al., 2019; Zhang et al., 2020). Different from the quasi-sterile lignocellulosic substrate to cultivate wood-decaying mushrooms such as *Pleurotus ostreatus* and *Lentinus edodes* (Chang and Hayes, 2013), the soil substratum compulsory for *M. importuna* fructification is an outdoor ecosystem with natural microbiota. Indeed, previous studies provided some clues that soil microbiota associated with morel cultivation could affect the outcome of morel fructification (Benucci et al., 2019; Zhang et al., 2019). It is hypothesized that the morel mycelium and associated microbial cortege metabolize and accumulate specific nutrients in the substratum, and the accumulated nutrients were consumed later to support morel fructification. The intrinsic C nutrients harbored by the organic matters in the soil substratum might also have a contribution to the morel fructification. However, the details about this process remain unknown.

Soil is a complex matrix with high complexity and randomness in its texture, density, granular size, spatial heterogeneity, and background microbiota (Aleklett et al., 2018). As a result, naturally developed soil is a challenging habitat to track the process of morel fructification. Since soil is composed of inorganic fractions such as minerals and rocks, plus organic matters including deeply decomposed biomass, it is possible that the morel could merely demand an inorganic matrix acting as a mechanistic carrier, plus some decomposed organic matters to supply essential nutrients and a cortege of microbiota to drive the fructification. A paradigm of “build your own soil” as proposed by Aleklett et al. (2018) inspired us that building a simpler and more controllable system by using a semi-synthetic substratum instead of ordinary soil might be a feasible way. Hydroponic substrata of synthetic or semi-synthetic origin, such as vermiculite or pearlite impregnated with Hoagland liquid medium (Van Der Straeten et al., 2001), have proven effective in the studies of plant nutrition and physiology. A similar strategy may apply to mushrooms by supplementing nutrients and microbiota into a non-degradable inorganic matrix. It has been observed that morel farms supplemented with some brands of composts can substantially increase the yield of fruiting bodies, while some other brands show slight or even adverse effects. It means that different types of composts might drive divergent patterns of microbial community succession and nutritional metabolism in the mushroom-bed substratum, which is possibly linked to different yield levels of morel fructification. This study used quartz particles as an inorganic matrix, and then blended fermented compost into the matrix to mimic the role of the organic matters and microbiota in the soil. Microbial community succession was followed, in combination with the metabolism of nutritional substances assayed. The patterns between different types of semi-synthetic substrata were compared, aiming to identify the key ecophysiological factors driving morel fructification.

## Materials and Methods

### Experimental Design

Two types of composts were used, respectively. One compost (C1) has proven very effective for years to increase the yield of black morel according to the feedback from local morel farmers. At the same time, the other (C2) was reported to have no positive effect. Composts C1 and C2 were blended with quartz particles respectively to form two different types of semi-synthetic substrata. Besides, a substratum with no compost supplemented (NC) was set by using the quartz particles solely. The three substrata (NC, C1, and C2) were paved in stainless-steel trays to grow *M. importuna* (Figure 1C). Each kind of substratum had three parallel trays as three individual replicates. The succession of morel mycelium together with the associated microbiota were surveyed by microbiome metabarcoding. The transformation and utilization of nutritional substances from the substratum was investigated with biochemical assays.

### Supplemented Composts

Composts C1 and C2 were purchased from the Xiaomei-Family Farm Co., Ltd., Jianyang, China, a small enterprise. Both composts were produced with two-stage procedures. They shared the same procedures in the first stage but had different ripening techniques for their second stages. In the first stage, a starting compost was made by 47.65% (w/w) of wheat straw (chopped to segments around 2 cm × 0.5 cm), 47.65% (w/w) of chicken manure, 1.50% (w/w) of rapeseed meal, 0.50% (w/w) of urea, 0.80% (w/w) of calcium biphosphate, 1.00% (w/w) of lime, 0.60% (w/w) of gypsum, 0.15% (w/w) of magnesium sulfate and 0.15% (w/w) of potassium sulfate. The ingredients were blended and filled in a solid-fermentation tank of 55 m length, 2.7 m width, and 0.8 m height. The compost was fermented for a total of 30 days and turned over each day with an excavator. It was a facultative anaerobic fermentation environment. Most of the time, during the fermentation of the first stage, the temperature inside the compost was around 65°C. After the first-stage composting, the mixture was divided into two parts as C1 and C2, for further ripening (Figure 1C). To make C1, the mixture was transferred to an open tank with a water heater in the shell. The tank was a lying half-cylinder and had stirring blades inside. Every hour the stirring blades were rolling for 5 min at a speed of 30 rpm. It was a relatively aerobic process. The tank was maintained at 65°C for 8 days and then turned to 80°C for an additional 2 days. To make C2, the mixture was loaded into an aerating cell of 4 m length, 3 m width, and 2 m height. The aerating cell was commonly used for composting the substratum for button mushroom (*Agaricus bisporus*). The compost was fermented under a constant aerating condition for 12 days with a ventilating rate of 10 m^3^ per minute. The temperature raised to 70°C on the second day, maintained for a week, and then dropped naturally to 40°C near the end of this period. The compost was then blended with an excavator and re-filled into the aerating cell to ripe for an additional 18 days. Every hour the compost was aerated for 10 min. The temperature inside raised again to 60°C and then dropped naturally to 30°C at the end of this period. As a final proceeding, both C1 and C2 were dried to a moisture level below 10% (w/w), homogenized and sieved.

### Preparation of the Semi-Synthetic Substratum

Quartz (silicon dioxide) particles with a purity grade of analytical reagent (AR) were purchased from Kelong Chemical Inc. (Chengdu, China). The quartz particles, with a diameter range between 0.18 and 0.25 mm, were heat-sterilized by the manufacturer to minimize the potential presence of background microbiota. Compost (C1 or C2) was blended into the quartz particles at a proportion of 2% (w/w). Within each compost type, the three replicates came from the same batch of compost. Each 100 kg of the mixture was evenly filled into a ventilated rectangular stainless-steel tray (90 cm by 80 cm). The substratum mixture paved in the tray was in a thickness of around 5 cm.

### Morel Cultivation

A diploid culture of the *M. importuna* SCYDJ1-A1 strain widely adopted in commercial agriculture was used in this study. Background information about this strain was detailed in previous work (Tan et al., 2019). Inoculant of the *M. importuna* SCYDJ1-A1 was prepared by culture amplification in three tiers: the so-called maternal spawn, proto-spawn, and cultivating spawn, respectively. The maternal spawn and proto-spawn were prepared following the procedures described previously (Tan et al., 2019), while the cultivating spawn used for sowing was grown on a spawn-substrate consisting of rapeseed straw shred (60% w/w) and wheat grains (40% w/w) (Miao et al., 2020) to exclude any soil from the ingredients. Granules of *M. importuna* SCYDJ1-A1 sclerotia in the cultivating spawn were mixed thoroughly and then sown evenly to the semi-synthetic substratum with 1 cm buried depth. The sowing date of this study was November 20th, 2018. Making and placing the aboveground nutritional supplementation (the so-called exogenous nutrient bags), field management, and fruiting-body harvest were carried out following the previously instructed procedures (Tan et al., 2019). Five exogenous nutrient bags with a total dry weight of 1.0 kg were placed in each tray onto the substratum surface. The trays were placed in an outdoor greenhouse of the Xiaomei-Family Farm (30.5°N, 104.5E), of which the illumination intensity at noon was around 2500–3500 lx, air temperature below 22°C, air relative humidity around 60–85%, oxygen percentage above 20.2% (v/v) and CO_2_ concentration below 500 ppm. The moisture in the semi-synthetic substratum detected by electronic sensors was maintained at around 20% during morel cultivation, while the temperature inside the substratum was about 6–13°C (Tan et al., 2021). The morel mycelium colonized the substratum (the so-called mushroom-bed), and the exogenous nutrient bags were placed onto the substratum 15 days after sowing to allow the morel mycelium colonizing the inside of the exogenous nutrient bags, thereby acquiring organic C nutrients from the stuffing consisting of wheat grains and rice husks (Tan et al., 2019).

### Sampling

Samples were collected on day 0 (November 20th, 2018), day 45 (January 4th, 2019), day 90 (February 18th, 2019), and day 135 (April 4th, 2019) after the morel-spawn sown to the semi-synthetic substrata. The morel fructified and was then harvested between day 90 and 135. A sterile plastic pipe with an inner diameter of 0.5 cm was pierced through the substratum and lifted to take a column of the full thickness of the substratum. Twenty columns were randomly sampled from each replicating tray and combined as a single sample. The three types of substrata, each possessing three parallel replicates, generated 3 × 3 replicate samples. The samples were collected in sealed vials and snap-frozen by liquid-nitrogen, stored at −80°C until used.

### Metabarcode Sequencing of Microbiome

Microbiome DNA in the semi-synthetic substratum was extracted using a CTAB extracting method (Tan et al., 2013). The solutions for the DNA extraction were freshly made, autoclaved, and kept sterile until used. Autoclaved pure water and quartz particles were used as two types of blank extraction controls. The blank extraction controls and the formal samples were extracted in the same batch using the same solutions of the CTAB extraction method to guarantee no microbiome contamination in the extraction reagents, as recommended by Hornung et al. (2019) and Zinger et al. (2019). Primers 515F (5′- GTGCCAGCMGCCGCGG -3′) and 907R (5′- CCGTCAATTCMTTTRAGTTT -3′) (Jiang et al., 2017) were used for amplification of the V4-V5 region of bacterial 16S rRNA gene, while ITS1-F (5′- CTTGGTCATTTAGAGGAAGTAA -3′) and ITS2-R (5′- GCTGCGTTCTTCATCGATGC -3′) (French et al., 2017) were used for fungal internal transcribed spacer (ITS) region. PCR amplicons were linked with index barcodes using the NEBNext Ultra DNA Library Prep Kit for Illumina (NEB, United States), following the manufacturer’s instructions to generate sequencing libraries. The samples of the blank extraction controls obtained no detectable yield of DNA extracts, and generated no sequencing library of metabarcoding amplicons by the PCR amplification. It confirmed that the DNA extraction and library construction steps introduced no contamination of microbiome.

The libraries were sequenced on an Illumina MiSeq platform at the sequencing facility of Biozeron Biological Technology Co., Ltd. (Shanghai, China) according to standard Illumina protocols. Paired-end reads were quality-controlled, merged by overlapping, and analyzed with the QIIME pipeline (Caporaso et al., 2010). Chimeras were removed by the USEARCH tool using the UCHIME algorithm (Edgar et al., 2011). Chimeras of 16S amplicons were removed with a combination of the *de novo* and reference methods against the Gold database^1^. Chimeras of ITS amplicons were removed with the *de novo* method.

### Bioinformatic Analysis of Microbial Community

Operational taxonomic units (OTUs) of bacteria and fungi were clustered with a similarity threshold at 97% using the UPARSE algorithm (Edgar, 2013). Bacterial OTUs were clustered against the SILVA reference database (Release 132), while fungal OTUs were clustered against the UNITE v7.2 (Full UNITE + INSD datasets) as described previously (Tan et al., 2019). The 16S OTUs were assigned to bacterial taxa using the RDP classifier of the QIIME pipeline, while the ITS OTUs were assigned to fungal taxa using the CONSTAX classifier (Gdanetz et al., 2017), as described by Longley et al. (2019). The sequencing depth was normalized among different samples. The number of assembled sequences in each sample that can be mapped to the OTU table was counted. The sample with the lowest number of sequences mapping to the OTU table was chosen as a subsampling criterion. The sequences of the other samples were all rarefied toward the subsampling criterion. Finally, the 16S amplicons of each sample were normalized to 31255 sequences, while the ITS amplicons were normalized to 29909. The OTU tables of the fungal and bacterial communities are provided in the online Supplemental Material (Supplementary Table 1). The coverage of OTUs in the microbial community was estimated by the rarefaction curve of OTUs (Supplementary Figure 1) as well as the Good’s Coverage index (Supplementary Table 2). The formula to calculate the Good’s Coverage index is *C* = 1-(*n*_1_/*N*), in which the *n*_1_ means the number of OTUs with only one sequence, and the *N* means the total number of sequences in the sample, as described at http://mothur.org/wiki/coverage/. OTUs with only one sequence (singleton OTUs) were removed prior to further statistical analysis.

The taxonomic richness of bacterial and fungal taxa was assessed by the ACE and Chao1 richness indices, while the community diversity was assessed by the Shannon-Wiener and Inverse Simpson’s diversity indices, as used previously (Tan et al., 2019). β-diversity of the communities in different samples was estimated by Non-metric Multi-Dimensional Scaling (NMDS) analysis using Bray-Curtis distance. The OTU table was normalized using cumulative sum scaling prior to calculation of the Bray-Curtis distance matrix, as described by Longley et al. (2019). Dispersion of community groups was estimated by permutational multivariate analysis of variance (PERMANOVA), using the “adonis” command in the R package vegan (Oksanen et al., 2016). The model formula for the PERMANOVA is: formula = as.dist(qiime.data$distmat) ∼ qiime.data$map[[opts$category]]. The differential distribution of taxa among the samples (biomarker taxa) at all taxonomic levels was determined by linear discriminant analysis effect size (LEfSe) as described by Segata et al. (2011), using the online tool LEfSe^2^. Biomarker taxa of the tested samples were judged with a linear discriminant analysis (LDA) score (log10) > 4.0 and *P*-value < 0.05. Ecological guilds and trophic modes in the fungal communities were predicted with FUNGuild using the online interface at https://github.com/UMNFuN/FUNGuild (Nguyen et al., 2016). Ecological functions of the bacterial communities were predicted with FAPROTAX version 1.2.4 (Louca et al., 2016).

The relationship between microbial community profiles and nutrient metabolism was estimated by the Redundancy Analysis (RDA), which is a non-parametric multivariate analysis of ecological data (Ramette, 2007), with Envfit test (Oksanen et al., 2016) provided by the R package vegan to estimate the significance of correlation. The proposed model is that the composition of microbial community (in particular, the phylum composition of bacterial community) around the time-points of morel fructification would affect potential transformation of C and N nutrients in the substratum to the biomass of morel ascocarps (yield). Therefore, the relative abundances of the bacterial phyla around the fructification period (average values of day 90 and 135) were treated as explanatory variables, and were sorted as a feature table, fitting onto the profile of consumed amounts of the tested C and N nutrients and the yield of morel as the response variables. The data table of relative abundances of bacterial phyla was subjected to the Hellinger’s transformation (Ramette, 2007), while the table of nutrient consumption and morel yield was subjected to the Z-score transformation, in order to normalize the values prior to the analysis.

### Assays for Biochemical Composition

Chemical components in the semi-synthetic substratum were quantified with classical analytical methods based on spectrophotometry or chromatography commonly used in soil geochemical determination. Specifically, total organic C was determined by a combustion method, while the samples were pre-treated with H_2_SO_3_ to remove inorganic carbonate, as described by Aye et al. (2016). Total N was determined from the H_2_SO_4_-digested sample by the micro-Kjeldahl method (Jackson, 1973). Ammonium N (in NH_3_ or NH_4_^+^ form) was determined using the micro-Kjeldahl method procedures without digesting the sample. N in nitrate form was determined with the salicylic acid colorimetric method described by Tian et al. (2008). Total P was determined by the classical Vanado-molybdophosphate spectrophotometric method (Tabatabai and Bremner, 1969) after the sample was digested and oxidized by HNO_3_-HClO_4_ to convert all P into phosphate. Available P was extracted with 0.5 mol l^–1^ NaHCO_3_ (pH 8.5) and then determined by the Vanado-molybdophosphate spectrophotometric method. Total K was determined by flame atomic absorption spectrometry (Ward et al., 1969). Available K was extracted with ammonium acetate and then determined by flame atomic absorption spectrometry. Humic substances were quantified using a modified Lowry method described by Guo et al. (2016).

Total proteins were determined with Pierce BCA Protein Assay Kit (Thermo Fisher Scientific, United States). Free amino acids were determined using the L-Amino Acid Quantitation Kit (Sigma-Aldrich, United States) based on oxidative deamination by L-amino acid oxidase. Total carbohydrates were determined from sulfate-digested samples using the Total Carbohydrate Assay Kit (Sigma-Aldrich, United States) based on the phenol-sulfuric acid method. Total soluble sugars were extracted with a boiling-water bath from undigested samples and then quantified using the Total Carbohydrate Assay Kit. After removal of readily soluble small-molecular saccharides by soaking (Tan et al., 2019), macromolecular structural carbohydrates (cellulose, β-glucan, and hemicellulose) were determined with the HPLC analyses described in the National Renewable Energy Laboratory (NREL) standard method (Sluiter et al., 2008). Lignin was extracted and quantified with the procedures described in the NREL standard method. Ether-soluble total lipids, commonly known as crude fats, were extracted with the Soxhlet extraction method described by Zhang et al. (2017), to represent the amount of total lipids. From the ether-extracted total lipids, triglycerides were determined using the Triglyceride Quantification Kit (Sigma-Aldrich, United States) based on colorimetric quantification of glycerol released from triglycerides, and free fatty acids were determined using the Free Fatty Acid Quantitation Kit (Sigma-Aldrich, United States) based on enzymatic oxidation of free fatty acids. Total phospholipids were estimated by determining the total P element content in the total lipids using an improved method modified from the American Oil Chemists’ Society (AOCS) Official Method Ca 12–55 (Chen et al., 2014). The method ashes the lipids with zinc oxide, and the residual is reacted with sodium molybdate to form a blue phosphomolybdic complex that can be quantified spectrophotometrically.

### Assays for Enzymatic Activity

Activities of key enzymes related to turnover of C, N, and P in the semi-synthetic substratum were measured with biochemical assays. Cellulase activity, covering endo-cellulase (EC 3.2.1.4) and exo-cellulase (EC 3.2.1.91 and EC 3.2.1.176), was measured by monitoring the release of free glucose from microcrystalline cellulose with the 3,5-dinitrosalicylic acid (DNS) method used by Zhu et al. (2013). Xylanase activity (EC 3.2.1.8), chosen to represent the potential of hemicellulose hydrolysis, was measured by monitoring the release of free xylose from birchwood xylan as described previously (Tan et al., 2018). Laccase activity (EC 1.10.3.2) was measured with the classic colorimetric method described by Touahar et al. (2014), which monitors the oxidation of 2,2’-azino-bis-(3-ethylbenzthiazoline-6-sulfonic acid) (ABTS) by optical absorbance at 420 nm. Peroxidase activity, covering manganese peroxidase (EC 1.11.1.13), lignin peroxidase (EC 1.11.1.14), and versatile peroxidase (EC 1.11.1.16) that cooperatively degrade lignin, was measured using L-DOPA as a substrate in the presence of peroxide (Talbot et al., 2015). Lipase activity (EC 3.1.1.3) was used to estimate the potential of the microbiota to utilize lipids in the substratum. It was measured with the Triglyceride Quantification Kit (Sigma-Aldrich, United States), which monitors the hydrolysis of triglycerides based on colorimetric quantification of glycerol released from triglycerides. Fatty-acyl-CoA synthase activity (EC 2.3.1.86) was used to estimate the potential of the microbiota to synthesize and accumulate lipids in the substratum. It was measured by monitoring the consumption of NADPH with the method described by Lynen (1962), which uses malonyl-CoA as the substrate. Total protease activity was measured with the Protease Fluorescent Detection Kit (Sigma-Aldrich, United States), which uses casein labeled with fluorescein isothiocyanate (FITC) as the substrate to estimate the potential of peptide-bond cleavage. Nitrifying and denitrification potentials were both measured using the procedures described by Chen et al. (2015). Phosphatase activity was measured by the classical Vanado-molybdophosphate colorimetric method (Tabatabai and Bremner, 1969), which monitors the release of free inorganic phosphate from *para*-nitrophenyl phosphate (pNPP) as an assay substrate. Tris–HCl buffer was used instead of phosphate buffer to avoid background P interference.

### Abundance Estimation of Functional Groups in N-Metabolism

*nifH* gene was used to estimate the abundance of N-fixation bacteria in the substratum with the primer pairs *nifH*-polF (5′- TGCGAYCCSAARGCBGACTC -3′) and *nifH*-polR (5′- ATSGCCATCATYTCRCCGGA -3′) (Poly et al., 2001). Bacterial *amoA* gene was used to estimate the abundance of ammonia-oxidizing bacteria (AOB) with primer pairs *amoA*-1F (5′- GGGGTTTCTACTGGTGGT -3′) and *amoA*-2R (5′- CCCCTCKGSAAAGCCTTCTTC -3′) (Rotthauwe et al., 1997). *nirK* and *nirS* genes encoding for bacterial nitrite reductases were used to estimate the abundance of denitrifying bacteria (Kandeler et al., 2006). Primers *nirK*-876F (5′- ATYGGCGGVCAYGGCGA -3′) and *nirK*-1040R (5′- GCCTCGATCAGRTTRTGGTT -3′) (Henry et al., 2004) were used to amplify the *nirK* gene, while *nirS*-Cd3aF (5′- AACGYSAAGGARACSGG -3′) and *nirS*-R3cd (5′- GASTTCGGRTGSGTCTTSAYGAA -3′) (Throback et al., 2004) were used to amplify *nirS*. The DNA copy numbers of the examined genes were determined on a CFX-Connect real-time PCR system (Bio-Rad, United States) with a 2xTaqMan Fast qPCR Master Mix Kit (Sangon Biotech Inc., Shanghai, China), following the detailed procedures described by Kandeler et al. (2006).

### Statistical Analysis

Significance of difference among three or more groups of samples was judged by the one-way ANOVA at *P*-value < 0.05, with Tukey HSD for false discovery rate correction in multiple testing. Significance of difference between two groups was judged by the *t*-test at *P*-value < 0.05. The PASW Statistics version 18 (IBM SPSS Inc., United States) software was used for the statistical tests.

### Accessibility of Strain and Data

*Morchella importuna* SCYDJ1-A1 culture can be obtained on request (for non-commercial research only) from both the Culture Collection Center of the Soil and Fertilizer Institute, Sichuan Academy of Agricultural Sciences, and the Jindi-Tianlingjian Co., Ltd., Sichuan, China. Metabarcoding datasets of bacterial 16S rDNA V4-V5 and fungal ITS are accessible under NCBI Bioproject PRJNA669301 and PRJNA670291, respectively.

## Results

### Morel Yield Driven by Different Composts

The morel fruiting bodies harvested from the substratum supplemented with C1 had a dry-weight yield of 102.10 ± 10.53 g m^–2^. It was the highest level among the three types of substrata (*P* < 0.001) (Figure 1C). It means that the nutrients and microbial ecology of C1 could support fructification effectively. The dry-weight yield from the C2 substratum was 0.54 ± 0.21 g m^–2^, which was a low yield comparable with (*P* = 0.490) the substratum of bare quartz particles with no compost supplementation (NC) (11.78 ± 4.30 g m^–2^). The composition of major nutritional substances in the fruiting bodies did not differ significantly among the three types of substrata (Supplementary Table 3).

### Microbial Community Shift

#### Community Diversity

**FIGURE 2:**
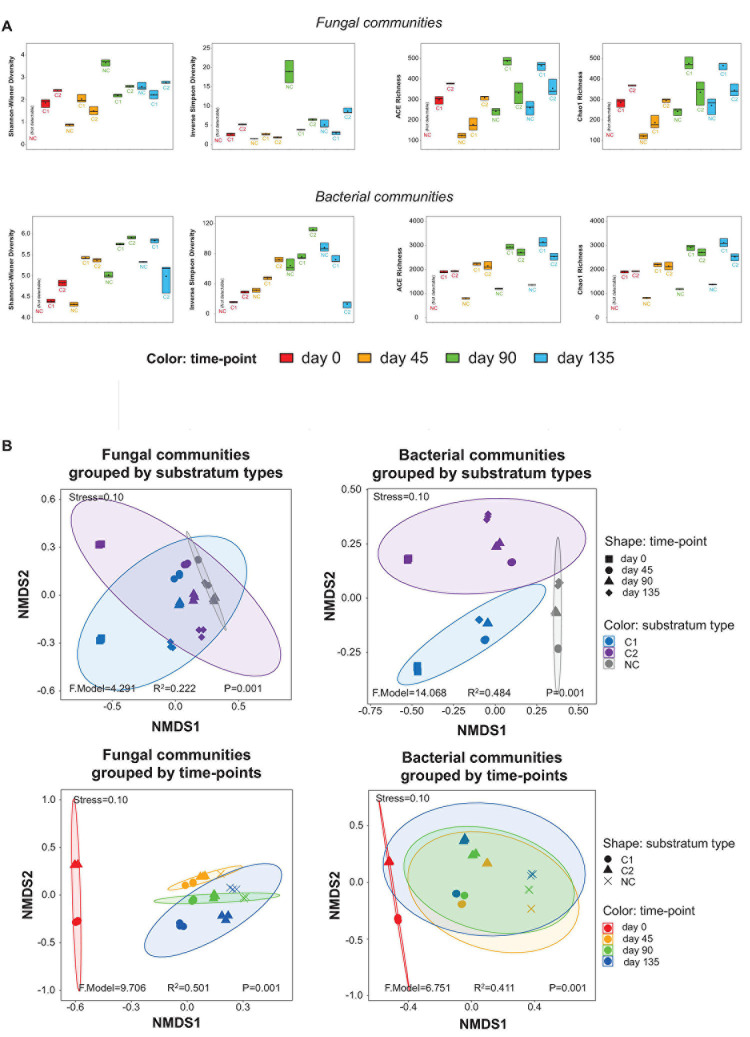
**(A)** α-diversity estimators of the fungal and bacterial communities, shown by boxplots. The values of the estimators are provided in the online Supplementary Materials as Supplementary Table 4. **(B)** Impacts of substratum type and time-point on the grouping of the fungal and bacterial communities, shown by NMDS plots based on the β-diversity of the samples.

**FIGURE 3:**
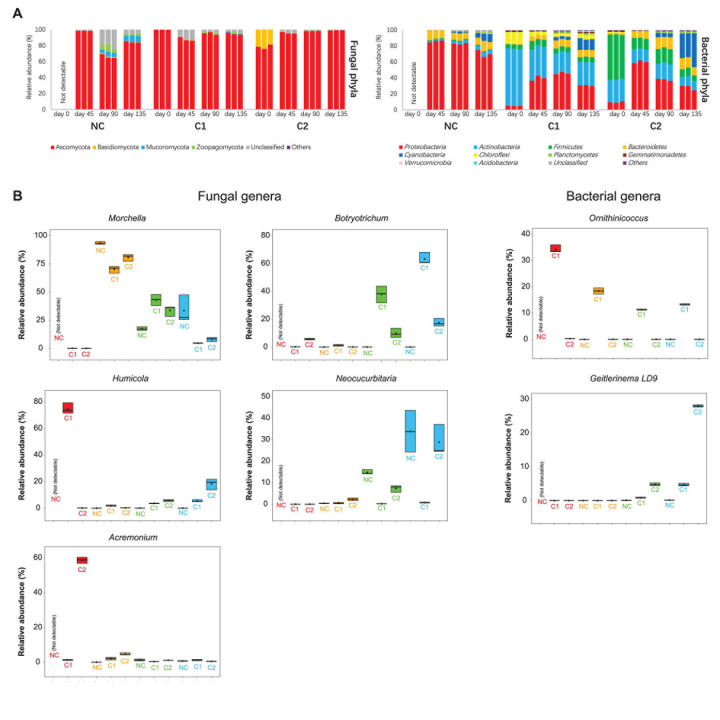
**(A)** Relative abundances of fungal and bacterial phyla in the NC, C1, and C2 substrata. **(B)** Relative abundances of important genera in the fungal and bacterial communities. Heatmaps showing more fungal and bacterial genera are in the Supplementary Figure 2.

Fungal and bacterial communities were surveyed at day 0, 45, 90, and 135 after the morel spawn sown into the semi-synthetic substrata, representing four key time-points of microbiota development during morel cultivation: starting microbiota, microbiota under the influence of aboveground nutritional supplementation, microbiota ready to support morel fructification, and microbiota at the end of the fructification. The fungal ITS region and the bacterial 16S V4–V5 region were used for metabarcoding of the fungal and bacterial communities, respectively. At the beginning of morel cultivation, each of the C1 and C2 substrata contained microbial communities introduced by the fermented composts. Since the sterilized quartz particles contained a sparse microbiota, the extraction of total microbial DNA from the NC substratum at day 0 generated no detectable DNA products, as expected. The PCR amplification using the metabarcoding primers also failed to generate any DNA products. The samples of NC at day 0 were therefore lacking and labeled as “not-detectable” (Figures 2A, 3A,B).

The taxonomic richness was estimated by the ACE and Chao1 richness indices, while the α-diversity was estimated by the Shannon-Wiener and Inversed Simpson diversity indices. The fungal and bacterial communities showed similar patterns of time-course shifts in taxonomic richness and α-diversity (Figure 2A). After day 45, the NC substratum was colonized by environmental microbial intruders, thereby establishing its microbiota naturally, but with lower levels of taxonomic richness than the substrata with composts. The three substrata showed very different patterns of α-diversity. In the NC substratum, the diversity of fungal and bacterial communities was initially low at day 45, reached a high peak at day 90, and then fell back at day 135. The C1 substratum showed the lowest fluctuation in the microbial diversity across the entire course. The C2 substratum showed a decreasing trend of microbial diversity during day 0–45 and then an increasing trend across day 45–135. Comparing the diversity at the same time-points among the three types of substrata, the results show that the bare-quartz (NC) substratum at day 45 had lower bacterial diversity than the two substrata supplemented with composts but reached unexpectedly high fungal diversity at day 90. Around the fructification and harvest period (day 90–135), the C1 substratum showed lower diversity of fungal and bacterial communities than the C2 substratum and the bare quartz particles.

Variation in the fungal and bacterial communities was estimated by β-diversity based on NMDS analysis (Figure 2B). The stress values of the NMDS plots at 0.10 indicate that the plots are valid. The samples were grouped by different types of substrata, and then grouped by the four time-points, respectively. The *P* values (all = 0.001) indicated that the groupings by substratum types and by time-points were valid. For the fungal communities, the *R*^2^ value of the grouping by substratum types (0.222) is smaller than that of the grouping by time-points (0.501). It suggests that stages during morel cultivation (time-points) were a bigger driver than the types of supplemented composts (substratum types) to shape the fungal communities. For the bacterial communities, an opposite trend was observed, although the *R*^2^ value of the grouping by substratum types (0.484) is close to that by time-points (0.411). It suggests that the types of supplemented composts had a slightly higher impact than the different stages of morel cultivation in shaping the bacterial communities.

#### Taxonomic Composition

The fungal communities of all the three types of substrata were dominated by an overwhelmingly high abundance of Ascomycota (all above 65%) during the entire course of morel cultivation (Figure 3A). The starting fungal communities of C2 contained 21% of Basidiomycota, but the abundance was not maintained afterward. At the genus level, the inoculated *Morchella* had very low abundance on the day of sowing. It increased rapidly to around 70–90% at day 45 (Figure 3B), which was the peak level during the entire course, as activated by the abundant C nutrients acquired from the exogenous nutrient bags. In the substratum supplemented with C1, *Morchella* shrank to an abundance around 40% at day 90. After the end of the morel harvest (day 135), the abundance further declined below 5%, reflecting exhaustion of mycelium biomass of the morel. This exhaustion did not necessarily link to a high yield of morel ascocarps aboveground since the C2 substratum showed a similar decline of *Morchella* abundance but obtained a very low yield. It means that the C2 substratum might lack some other key factors to drive morel fructification. In the substratum of bare quartz (NC), the succession of *Morchella* abundance showed a different pattern. It fell below 20% at day 90 when it should have been the time to get ready for fructification, but increased again at day 135 to an average abundance of over 33%, for unknown reasons. Relative lack of nutrients in the NC substratum might be a reason leading to the unexpected fluctuation of morel mycelium abundance. The starting fungal communities of C1 were dominated by an overwhelmingly high abundance of *Humicola* (75%), while C2 was dominated by *Acremonium* (59%) (Figure 3B). The *Humicola* and *Acremonium* were also recognized by the LEfSe as the biomarker fungal genera of C1 and C2 at day 0, respectively (Supplementary Figure 3). The noticeable difference in the dominant fungi is likely a consequence of the different ripening techniques adopted by C1 and C2 in the second stage of composting. The dominant fungal taxa *Humicola* and *Acremonium* in the starting microbiota of C1 and C2 were neither sustained afterward.

When looking at the bacterial communities, C1 contained had much higher abundances of *Actinobacteria* (72%) and *Chloroflexi* (15%) in comparison to C2 (*P* < 0.001), while C2 was colonized by *Firmicutes* as the most abundant phylum (56%). This pattern is sustained to day 135 (Figure 3B). The LEfSe analysis also recognized *Actinobacteria* and *Firmicutes* as biomarker phyla for the bacterial communities of C1 and C2, respectively (Supplementary Figure 3). *Cyanobacteria* showed a rapid expansion from before to after morel fructification (day 90–135), in all the three types of substrata (NC: *P* = 0.001; C1 and C2: *P* < 0.001). It is the biomarker phylum of C2 after morel fructification (Supplementary Figure 3). At genus level, an actinobacterial genus *Ornithinicoccus* had a high abundance in C1 (>30%) at day 0. The high abundance lasted across the growth period of morel, although to a lesser extent during the period of day 45–135 (still > 10%). Some other actinobacterial genera *Glycomyces*, *JG30-KF-CM45_norank*, and *Actinomarinales_norank* in the C1 substratum showed similar trends (Supplementary Figure 2). In a different pattern, two major bacterial genera *Planifilum* and *Novibacillus* in the starting bacterial communities of C2, were difficult to sustain their high abundances afterward (Supplementary Figure 2). Altogether, the results show that supplementation of different composts established different composition and succession of microbiota in the substrata.

#### Predicted Fungal Guilds and Bacterial Functional Groups

**FIGURE 4:**
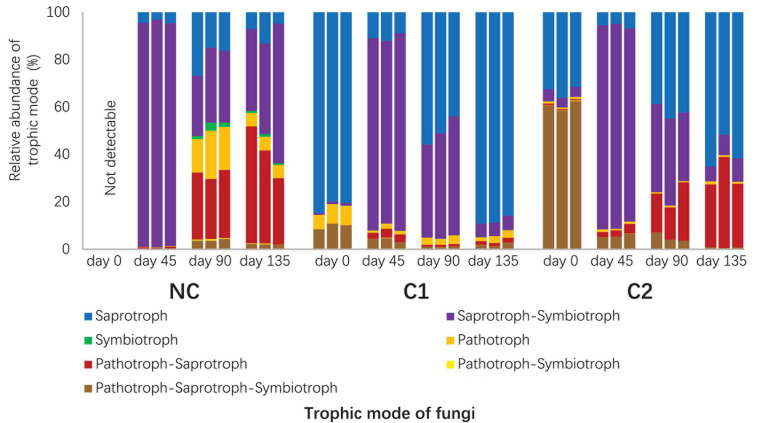
Relative abundances of trophic modes in the fungal communities predicted with FUNGuild. The unassigned portion was discarded.

A total of 1235 fungal OTUs were annotated for their trophic modes and ecological guilds by using FUNGuild. The 1235 fungal OTUs had 115 with a confidence ranking at highly probable, 547 at probable, and 344 at possible. 229 OTUs were not assigned to any guild (Supplementary Table 4). Since the *Morchella* and *Acremonium* with overwhelmingly high abundances were classified into the confidence ranking of probable and possible, respectively, we used all the predictions ranked as highly probable, probable, and possible. The fungal communities in the three substrata showed divergent composition of trophic modes and ecological guilds (Figure 4 and Supplementary Figure 4). The starting microbiota of C1 was dominated by an overwhelmingly high abundance of saprotrophs (Figure 4), *Humicola* being the dominant genus. In comparison, the initial microbiota of C2 contained fewer saprotrophs than the sum of pathotrophs and facultative pathotrophs (*P* < 0.001). *Acremonium*, predicted by FUNGuild as a facultative pathotroph–saprotroph–symbiotroph type, was the major pathotroph in the starting microbiota of C2 (Supplementary Table 4). The rapid proliferation of *M. importuna* mycelium at day 45 (Figure 3B) was the main reason for the overwhelmingly high abundance of the saprotroph–symbiotroph group in the three substrata. It was classified as a facultative saprotroph belonging to the ectomycorrhizal-undefined saprotroph-wood saprotroph guild (Supplementary Figure 4). When the morel mycelium was about to produce ascocarps (at day 90), the C1 substratum had a much lower abundance of the pathotroph–saprotroph group (0.93% ± 0.08%) than C2 (18.25% ± 5.73%, *P* = 0.002) and NC (27.46% ± 1.87%, *P* < 0.001). This trend lasted through the fructification process until the finish of harvest (*P* = 0.007 compared with C2; *P* = 0.002 compared with NC). The high abundance of the pathotroph–saprotroph in C2 and NC around the fructification period was mainly contributed by the plant pathogen-wood saprotroph guild (Supplementary Figure 4). It suggests that fungi of facultative pathotroph–saprotroph nutrition, such as *Neocucurbitaria* spp. (Supplementary Table 3), might have negative influences on the fructification yield of morel. When looking at the predicted functional groups related to N metabolism (Supplementary Figure 5), the results show that the starting microbiota inoculated by the C1 compost had a higher abundance of the nitrification group (*P* = 0.001) and a lower abundance of the denitrification group (*P* < 0.001) than C2. The predicted abundances of nitrification and denitrification bacterial groups had a similar trend with the distribution of gene copies involved in nitrification and denitrification functions estimated by qPCR (Supplementary Figure 6). The nitrate respiration and reduction groups that consume nitrate also had lower abundances in C1 than C2 and NC at both day 0 and 45 (all *P* < 0.01). From day 45 to 90, the C1 substratum maintained a lower abundance of the N-fixation group than C2 and NC (all *P* < 0.01). It is similar to the distribution of *Cyanobacteria* in the substrata (Figure 3A). These findings suggest that the microbiota from the C1 compost might facilitate an accumulation of nitrate in the substratum while limiting an excessive accumulation of ammonia.

### Metabolism and Accumulation of Nutrients

**FIGURE 5:**
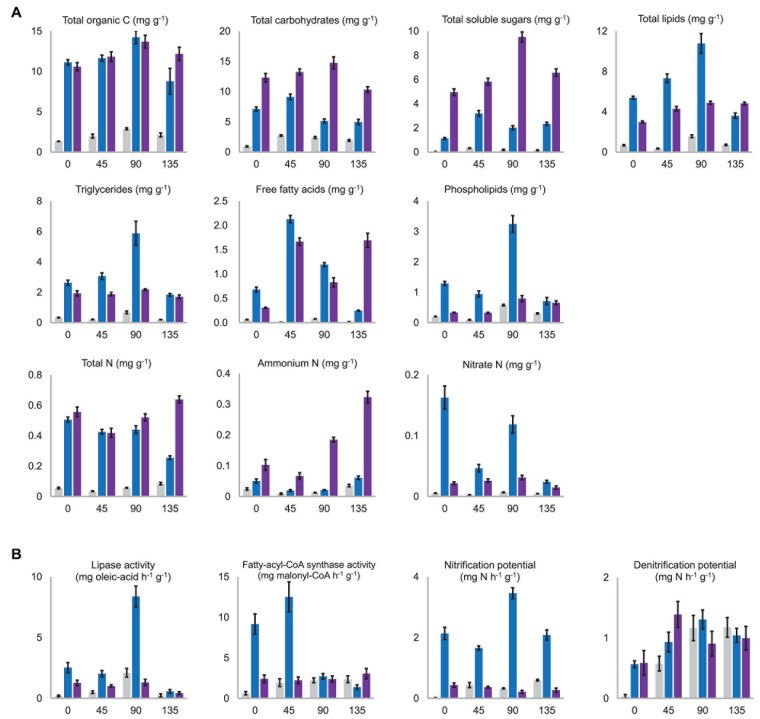
**(A)** Contents of the major nutritional substances which were significantly differentiated among the substrata. NC, C1, and C2 were colored in gray, blue, and purple, respectively. All the presented values are the mean of three biological replicates with standard deviation bars. The horizontal axis in all charts means days after morel sowing. A complete version of this figure showing all the nutritional substances determined in this study is provided in the Supplementary Figure 7. **(B)** Activity levels of key enzymes for C and N metabolism, which were significantly differentiated among the substrata. The activity level was shown as the catalytic ability to transform one milligram of the assay-substrate per hour by one gram (dry weight) of the substratum. A complete version of this figure showing all the activities measured in this study is provided in the Supplementary Figure 8.

The C1 and C2 substrata both provided diverse arrays of C and N nutrients (Supplementary Figure 7). The content of total organic C was around 11 mg kg^–1^, similar between the C1 and C2 substrata, from the beginning (day 0) (*P* = 0.238) until the time-point when the morel was about to produce fruiting bodies (day 90) (*P* = 0.591) (Figure 5A). From day 90 to 135, around 5.64 mg kg^–1^ of the organic C content was consumed in the C1 substratum. It means that the fructification process significantly consumed the organic C content in the C1 substratum (*P* < 0.001). In comparison, the organic C content in C2 was similar between day 90 (13.70 ± 0.79 mg kg^–1^) and day 135 (12.19 ± 0.82 mg kg^–1^) (*P* = 0.105), indicating few organic C nutrients consumed in C2. It is in line with the high yield from the C1 substratum and the low yield from C2, suggesting that the organic nutrients contained in the substrata were converted to the biomass of fruiting bodies. The content of humic substances increased during the period of day 45–135, not only in the two substrata supplemented with composts but also in the substratum of bare quartz (*P* < 0.001) (Supplementary Figure 7). It suggests that the decomposed biomass from morel mycelium together with the associated microbiota could eventually transform into inert organic matters, despite the background level of organic C in the system. Total P, available P, total K, and available K were similar between the C1 and C2 substrata at all the four examined time-points (all *P* > 0.05) (Supplementary Figure 7). Based on the same ingredients, the C1 and C2 composts tend to contain similar levels of P and K contents and led to similar dynamics of P and K fluctuations during the morel-cultivation course. Indeed, the microbiota of C1 and C2 showed a similar potential to mineralize P from the supplemented composts (Supplementary Figure 8).

As expected, the NC substratum consisting of pure quartz particles was in short of all kinds of nutritional substances compared with C1 and C2 (Figure 5A and Supplementary Figure 7). Its low level of nutrients at day 0 were likely brought by the sclerotia (the so-called morel spawn) sown into the substratum. The aboveground nutritional supplementation of exogenous nutrient bags likely increased the NC substratum’s organic C content before fructification (*P* < 0.001), but it was still a nutrient-limited substratum throughout the entire course of morel growth and fructification.

#### Carbohydrates and Lignin

Among specific fractions of the organic C nutrients, the C2 substratum maintained higher contents of total carbohydrates (*P* < 0.001) as well as total soluble sugars than C1 throughout the entire course (*P* < 0.001) (Figure 5A). β-glucan, including cellulose from plant debris in the composts as well as structural β-glucan building up fungal cell walls, had similar contents in the C1 and C2 substrata throughout the entire course (*P* = 0.606, 0.189, 0.546, and 0.347 at day 0, 45, 90, and 135, respectively) (Supplementary Figure 7). The peak content of β-glucan appeared around day 45, indicating an active proliferation of fungal biomass during this period. The decline of β-glucan from day 45 to 90 (*P* < 0.001) is partially due to cellulose decomposition in the composts by cellulases produced by the microbiota, while consumption of fungal biomass at the same time could not be precluded. The hemicellulose content was similar between the C1 and C2 substrata at the beginning (*P* = 0.262), while the microbiota in the C1 substratum seemed more efficient in degrading and consuming hemicellulose than C2 during the period before day 90 (*P* < 0.001). Lignin content showed decreasing trends in both the C1 (*P* = 0.005) and C2 (*P* = 0.002) substrata. It means that the microbiota in the substrata was decomposing the residual plant debris remaining in the composts. Estimated by the activities of cellulase, xylanase, laccase, and peroxidase, the microbiota of NC, C1, and C2 showed similar potential in lignocellulosic decomposition (Supplementary Figure 8).

#### Lipids

At the beginning of morel cultivation (day 0), the total-lipid content in the C1 substratum (5.39 ± 0.15 mg kg^–1^) was nearly twice that in C2 (2.98 ± 0.11 mg kg^–1^) (Figure 5A). It means that the C1 compost blended into the quartz particles had richer lipid nutrients than C2 (*P* < 0.001) and suggests that a large proportion of the organic C in the C1 compost was in the form of lipids. In addition to the richer lipids initially, a noticeable accumulation of lipids from 5.39 ± 0.15 mg kg^–1^ to 10.78 ± 0.98 mg kg^–1^ during the period before fructification (day 0–90) was observed in the C1 substratum (*P* < 0.001). It is in line with the high potential of fatty-acid biosynthesis in C1 as estimated by the fatty-acyl-CoA synthase activity (Figure 5B). A similar accumulation of lipids from 2.98 ± 0.11 mg kg^–1^ to 4.91 ± 0.15 mg kg^–1^ was observed in the C2 substratum, significant (*P* < 0.001), but to a lesser extent. The accumulation of lipids at day 45 was likely contributed by a rapid increase of free fatty acids during day 0–45 rather than triglycerides in both the C1 and C2 substrata (Figure 5A). Morel fructification during day 90–135 consumed 67% of the lipid stock in the C1 substratum, from 10.78 ± 0.98 mg kg^–1^ to 3.61 ± 0.26 mg kg^–1^. It is supported by the extraordinarily high level of lipase activity at day 90 (Figure 5B). Specifically, the consumption of triglycerides, free fatty acids, and phospholipids was all prominent in the C1 substratum during this period (*P* < 0.001). In comparison, no significant consumption of lipids was observed in the C2 substratum during day 90–135 (*P* = 0.940). This corresponds to the high yield of morel fruiting bodies harvested from the C1 substratum and the low yield from C2. Interestingly, the content of free fatty acids in the C2 substratum increased during day 90–135, which might be generated from hydrolysis of triglyceride stock or from the conversion of other organic C nutrients.

In the NC substratum, due to lack of organic matters, the morel mycelium and associated microbiota seemed to have consumed a lot of the background lipids during the period of day 0–45 (*P* = 0.007), which were brought into the substratum along with the inoculation of morel spawn in the beginning. From day 45 to 90, a significant accumulation of lipids was also observed in the NC substratum (*P* < 0.001), although the amount was tiny (from 0.36 ± 0.05 mg kg^–1^ to 1.57 ± 0.14 mg kg^–1^) as compared to that in C1. Morel fructification in the NC substratum during day 90–135 also consumed a part of the lipid stock (*P* < 0.001), although at a low level (from 1.57 ± 0.14 mg kg^–1^ to 0.72 ± 0.07 mg kg^–1^). It corresponds to the low yield of fruiting bodies harvested from the NC substratum (11.78 ± 4.30 g dry-weight m^–2^).

#### Nitrogen

The C1 and C2 substrata had initial levels of total N at 505.56 ± 17.07 μg kg^–1^ and 557.35 ± 31.72 μg kg^–1^, respectively, which were quite close (*P* = 0.053) (Figure 5A). In the period of morel fructification and harvest (day 90–135), significant consumption of total N was observed in the C1 substratum (*P* < 0.001), while the C2 and NC substrata both showed an unexpected increase of total N content (*P* = 0.003 and 0.001, respectively). When looking at different fractions of N nutrients, the C1 substratum maintained more abundant nitrate N than the C2 and NC substrata across the four time-points (all *P* < 0.01). It is in line with the greater potential of nitrification (Figure 5B) as well as the higher abundance of *amoA* copies observed in C1 (all *P* < 0.01) (Supplementary Figure 6). Consumption of nitrate N was prominent during day 0–45 and again during day 90–135 (*P* < 0.001). Similar consumption of nitrate N was observed in the NC substratum, although to a lesser extent (day 0–45: *P* < 0.001; day 90–135: *P* = 0.002). In comparison, no consumption of nitrate N appeared to take place in the C2 substratum during day 0–45 (*P* = 0.331), while only a small amount (16.52 μg kg^–1^) was consumed during day 90–135 (*P* = 0.001). The C1 substratum maintained higher levels of nitrate N than ammonium N from day 0 to 90 (day 0: *P* = 0.001; day 45: *P* = 0.009; day 90: *P* < 0.001). In comparison, the C2 substratum maintained higher levels of ammonium N than nitrate N at the four time-points (day 0: *P* = 0.001; day 45: *P* = 0.003; day 90 and day 135: *P* < 0.001). It is likely due to the weak nitrification potential of C2 and NC that could hamper the transformation of ammonium toward nitrate (Figure 5B). Indeed, the microbiota in C2 and NC contained lower abundances of the ammonia-oxidizing gene (all *P* < 0.01) (Supplementary Figure 6). These results mean that nitrate N was the major form of inorganic N in the C1 substratum before fructification, while ammonium N was the major form in C2. Moreover, an increase of ammonium N from day 90 to 135 was observed in the three types of substrata (*P* < 0.001), and the increasing extent in C2 was particularly prominent (Figure 5A). It is in line with the expansion of *Cyanobacteria* in the microbiota during day 90–135 (Figure 3B), and is also supported by the increased *nifH* copies (Supplementary Figure 6). The proliferation of *Cyanobacteria* could lead to a more active N-fixation process, whose primary product is ammonium N. When looking at organic-N fractions, the C1 and C2 substrata showed similar consumption of proteins during the fructification period (*P* = 0.131) (Supplementary Figure 7). Free amino acids showed no significant consumption during the period in C1 and C2 (*P* = 0.129 and 0.099, respectively), and even an increase in NC (*P* = 0.004). It suggests that the stocks of proteins and free amino acids in the substratum were unlikely the primary fuel to supply the fructification.

### Fructification-Related Nutrient Metabolism Driven by Microbiota

**FIGURE 6:**
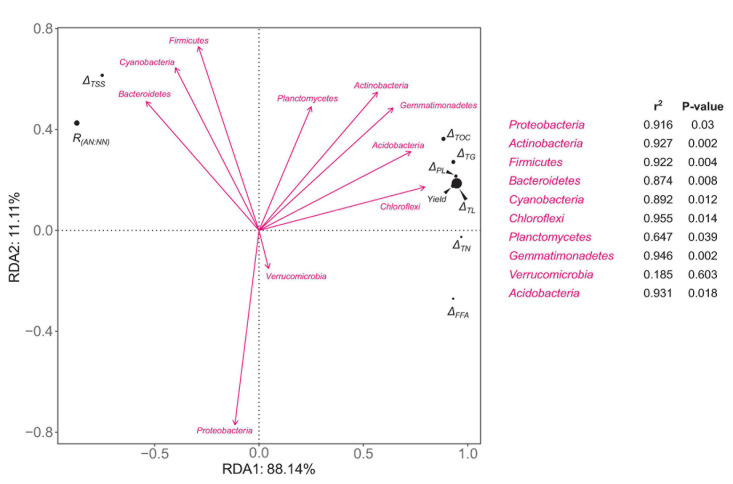
RDA plots of the relative abundances of bacterial phyla versus ecophysiological factors involved in fructification-related metabolism of C and N nutrients. The relative abundance of each bacterial phylum is an average value of day 90 and 135. Δ*_*T*__*OC*_*, Δ*_*T*__*SS*_*, Δ*_T__*L*_*, Δ*_*T*__*G*_*, Δ*_*PL*_*, Δ*_*F*__*FA*_*, and Δ*_*T*__*N*_* mean the consumed amounts (the difference between day 90 and 135) of total organic C, total soluble sugars, total lipids, total triglycerides, total free fatty acids, total phospholipids, and total N, respectively. *R*_(*AN:NN*)_ means the ratio of ammonium-N:nitrate-N. The strength of correlation is estimated by the length of arrow in combination with the *R*^2^ and *P* values calculated by Envfit test.

An RDA analysis was performed to estimate the influences of microbial community composition on the metabolism of important C and N nutrients during morel fructification as well as the yield. The relative abundances of bacterial phyla were chosen as the explanatory variables. For each phylum, the relative abundances at day 90 and 135 were transformed to an arithmetical mean to represent its average level during the fructification period. The consumed amounts (from day 90 to 135) of total organic C, total soluble sugars, total lipids, total triglycerides, total free fatty acids, total phospholipids, and total N, the ratio of ammonium-N:nitrate-N, and the yield of morel fruiting bodies, were chosen as the response variables. The RDA plots show that *Chloroflexi*, *Acidobacteria*, *Gemmatimonadetes*, and *Actinobacteria* were positively correlated with the consumption of total organic C, total lipids, triglycerides, and phospholipids (Figure 6). The consumption of these lipid-nutrients was strongly correlated with the yield of morel. In contrast, the consumption of total soluble sugars seemed negatively correlated with the yield. The results indicate the importance of C-source type for morel fructification. Moreover, the ratio of ammonium-N:nitrate-N was also negatively correlated with the yield. It again indicates that nitrate N seemed beneficial to the fructification, while ammonium N showed a negative influence. *Cyanobacteria* and *Bacteroidetes* seemed closely related with a high ratio of ammonium-N:nitrate-N, as well as a major C supply by soluble sugars instead of lipids, which were not optimum for a high yield of morel fructification.

## Discussion

Morels and truffles are among the few taxa in the Ascomycota that produce highly prized edible fungi from soil environments. The genetic and ecophysiological mechanisms involved in symbiosis and nutritional acquisitions of ectomycorrhizal truffles have been well studied (Murat et al., 2018). In contrast, morels with flexible lifestyles remain mysterious in their mechanisms of acquiring necessary nutrients from soils to fulfill their fructification (Liu et al., 2018). This study established a semi-synthetic system that simulates the process of morel scavenging essential nutrients from decomposed organic matters to support its fructification, which is analogous to the process taking place in soils. Compared with ordinary soils, the substratum adopted in this study by blending pre-sterilized quartz particles with compost is more controllable and reproducible in many physical, chemical, and biological factors.

By comparison, differentiated ecophysiological factors between the C1, C2, and NC substrata were identified, aiming to explain the divergent performances of fructification yield from the three substrata. Lipids were a major C source for the *M. importuna* mycelium and the associated microbiota in the C1 substratum, supported by the high activity of lipase (Figure 5B) in combination with the noticeable consumption of lipids during the fructification period (Figure 5A). Previous work reported that the morel mycelium colonizing the exogenous nutrient bags accumulated lipid nutrients *in situ* at the early stage, yet much fewer than carbohydrates, and then consumed the lipid stock afterward, while a disproportionately high activity of lipase was observed throughout the process (Tan et al., 2019). This hint suggested that lipids had the potential to become a major C source favored by the morel in a certain period. Indeed, the present study witnessed the relationship between morel fructification and lipid consumption in the C1 substratum. It also suggests a prospect of amending lipid-accumulating microorganisms to enhance the fructification yield of morel. In comparison, soluble carbohydrates were a major C source throughout the entire course in the C2 substratum (Figure 5A). The readily accessible C source supports enriched fungal guilds of pathotrophs and facultative pathotrophs. Comparative genomics revealed that fungi belonging to symbiotrophic and pathotrophic mode had lost a part of their oxidoreductases and CAZymes, leading to a preference of feeding on readily accessible C sources rather than scavenging from lignocelluloses (Kohler et al., 2015). Among the bacterial communities, the enrichment of fermentative consortia in the predicted functional groups of C2 is also related to the presence of readily accessible C sources. It suggests that agricultural managements targeted to reducing the contents of readily accessible C sources in semi-synthetic substratum as well as ordinary soils might benefit the yield performance of morel fructification.

N nutrients in the substratum are essential for morel fruiting (Tan et al., 2019; Zhang et al., 2020). However, different forms of N compounds could show divergent effects, either beneficial or inhibitory. Knowing that ammonia accumulation in *A. bisporus* substratum can adversely affect mycelium growth while favoring fungal parasites (Carrasco and Preston, 2020), the high amount of ammonium N in the C2 substratum showed a similar association with the proliferation of fungal and bacterial parasites in the microbiota as well as a low yield of morel fructification. It could even be aggravated by the rapid proliferation of N-fixation *Cyanobacteria* during the fructification period (Figure 3A). It suggests a prospect of amending chemical agents antagonizing *Cyanobacteria* in combination with ammonium-removing microorganisms such as ammonia-oxidizing archaea (AOA), AOB, comammox (Xia et al., 2018), and anammox (You et al., 2020) into morel agriculture to potentially enhance the performance of morel fruiting body yield.

The major bacterial phyla *Actinobacteria*, *Chloroflexi*, and *Proteobacteria* in the semi-synthetic substratum in this study (Figure 3A) were also dominant in the soil beneath the fruiting bodies of *Morchella sextelata*, another black morel species (Benucci et al., 2019). During the fructification period of *Morchella rufobrunnea* cultivated indoors, different profiles of bacterial communities were observed (Longley et al., 2019). Among the fungal communities, a high abundance of *Gilmaniella* seemed associated with a successful fructification of *M. rufobrunnea* cultivated indoors, while the dominance of *Cephalotrichum* seemed responsible for a failure of the fructification (Longley et al., 2019). This pattern is analogous to what happened in our present study. Indeed, certain pathotrophic fungal species have proven to inhibit the growth of morel mycelium via antagonistic exudates (Wang et al., 2020). It suggests that growing edible mushrooms relies on multiple microbial interactions, not only between bacteria and fungi (Carrasco and Preston, 2020) but also between microfungi and macrofungi.

Usage of semi-synthetic substratum in mushroom cultivation has very few examples. The dung saprotrophic Basidiomycete mushroom *A. bisporus* grows on a substratum of compost covered with a thin layer of casing soil (Pardo-Giménez et al., 2017). A facultatively saprotrophic morel species, the *M. rufobrunnea*, requests a substratum with a relatively higher proportion of compost (soil to compost at 50%:50% (v/v)) (Longley et al., 2019), while the semi-synthetic substratum for *M. importuna* in this study is mostly composed of quartz particles with no more than 2% (w/w) of organic-matter supplementation. Compared with the substrata used for *A. bisporus* and *M. rufobrunnea* dominated by organic-matters, the semi-synthetic substratum for *M. importuna* is more analogous to sandy soils. The determined key physiochemical characteristics (e.g., pH, total organic C, total N, available N, ammonium N, nitrate N, total P, available P, total K, available K, and humic substances) in the C1 and C2 substrata (Supplementary Figure 7) were within comparable ranges as those reported in ordinary agricultural soils (Chhabra et al., 2012; Griffiths et al., 2012; Fraser et al., 2015; Huang et al., 2019; Tan et al., 2019). The variations of physiochemical characteristics among these soils were even greater than those compared to the semi-synthetic substrata in this study. The high yield obtained from the C1 substratum (Figure 1C) indicates that the fructification of black morel was not necessarily dependent on real soils. It confirmed our expectation that morel merely demands an inorganic matrix acting as a mechanistic carrier, some decomposed organic matters to provide essential nutrients, as well as a cortege of microbiota to drive the fructification.

Morels cultivated in ordinary farmland soils have been reported to suffer from various soil-borne pathogens (He et al., 2017, 2018; Lan et al., 2020; Wang et al., 2020). Besides, morel farmers also reported many cases of severe decreases in morel yield that were suspected as relating to abuse of fungicide, herbicide, and pesticide. Compared with naturally developed soils, the semi-synthetic substratum is more controllable and reproducible in chemical composition and microbiota. When using the semi-synthetic substratum C1 as the mushroom-bed in this study, the fruiting body yield (Figure 1C) was slightly higher than using ordinary sandy-loam soil of a farm as reported previously [77.27 ± 3.38 g dry-weight m^–2^ (Tan et al., 2019), *P* = 0.081], and is comparable with the average yield level of large-scale production in farms (Liu et al., 2018). The nutritional quality of the fruiting bodies was also similar to those produced from ordinary agricultural soils (Tan et al., 2019, 2021). It might provide an alternative form of mushroom-bed for industrialized cultivation of black morel in the future to replace ordinary soils. Indeed, synthetic soil and soil-like substratum have definite advantages of higher controllability, reproducibility, and transparency of nutritional composition. They have great potentials in modern and future agriculture, such as facility cultivation and plant factory. However, the high economic cost of synthetic soil is an obvious drawback that hinders its wide application. Besides, turning industrial wastes into soils could raise concerns about potential contamination of heavy metals and other toxic substances (Marshall, 2007).

## Conclusion

**FIGURE 7:**
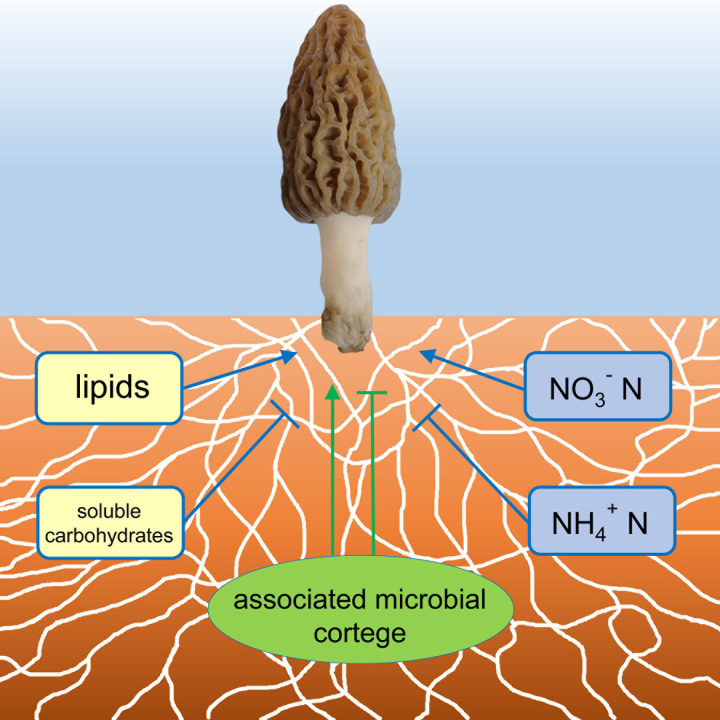
A conceptual illustration of the ecophysiological factors in the morel-cultivating substratum influencing the fructification. Factors with positive influences were indicated with arrows, while those with negative influences were indicated with T-terminal lines.

The study demonstrated a successful growing and fruiting course of the soil-saprotrophic black morel *M. importuna* in a semi-synthetic substratum, in which the morel mycelium colonizes the substratum with a cortege of microbiota (Figure 7), decomposes organic matter and accumulates lipids to supply the fructification. N nutrients are essential, while an appropriate form of nitrogenous substances is requested.

## Data Availability Statement

The datasets presented in this study can be found in online repositories. The names of the repository/repositories and accession number(s) can be found in the article/Supplementary Material.

## Author Contributions

HT: conceptualization, funding acquisition, investigation, supervision, and writing–original draft. YY: investigation and software. JT: resources. TL: data curation, formal analysis, and investigation. RM: investigation and methodology. ZH: funding acquisition. FM: validation, writing–review and editing, and supervision. WP: funding acquisition, project administration, and supervision. All authors contributed to the article and approved the submitted version.

## Conflict of Interest

The authors declare that the research was conducted in the absence of any commercial or financial relationships that could be construed as a potential conflict of interest.

## Footnotes

1 http://drive5.com/uchime/uchime_download.html

2 http://huttenhower.sph.harvard.edu/galaxy/root?tool_id=lefse_upload
