# Supplementary material for: Build Your Own Mushroom Soil: Microbiota Succession and Nutritional Accumulation in Semi-Synthetic Substratum Drive the Fructification of a Soil-Saprotrophic Morel
Source: Front Microbiol. 2021 May 24;12:656656. doi: 10.3389/fmicb.2021.656656 (PMC8180906; doi:10.3389/fmicb.2021.656656)
Supplement: Supplementary file 3 [file Data_Sheet_1.PDF]

Supplemental materials

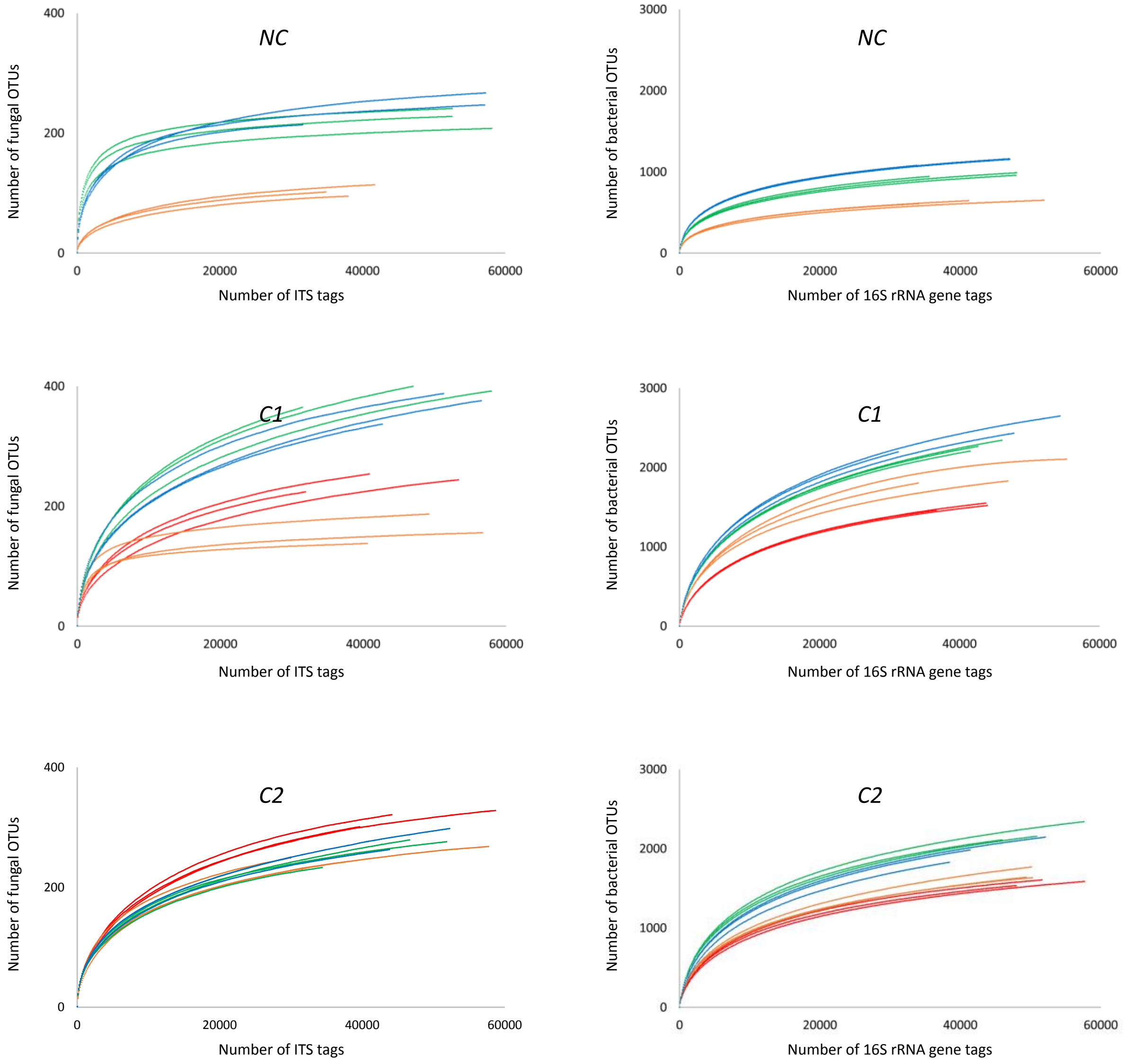

Figure S1 The rarefaction curves of the fungal and bacterial OTUs in the NC, C1, and C2 substrata. The samples at day 0, 45, 90, and 135 were colored in red, orange, green, and blue, respectively.

Table S1 OTU tables of the fungal and bacterial communities. The table is of big size and is therefore provided as multiple working-sheets in an individual Excel file available online: TableS1.xlsx.

Table S2 Diversity of the fungal and bacterial communities in the semi-synthetic substrata at day 0, 45, 90, and 135. OTUs were clustered at 97% similarity. Lower and upper limits of 95% confidence are presented in parentheses.

Community	Supplemented compost	Sampling time-point	Replicate	Observed OTUs	Community coverage	ACE richness	Chao1 richness	Shannon-Wiener diversity	Inverse Simpson's diversity
Fungi	NC	day 0	1	Not detectable					
			2						
			3						
		day 45	1	102	0.9993	125 (113,152)	122 (110,155)	0.89 (0.88,0.91)	1.5188 (1.5047,1.5333)
			2	95	0.9994	113 (103,135)	108 (100,133)	0.83 (0.81,0.84)	1.4797 (1.4669,1.4925)
			3	114	0.9994	135 (124,159)	133 (121,164)	0.92 (0.90,0.93)	1.5323 (1.5191,1.5458)
		day 90	1	241	0.9996	256 (247,275)	254 (246,279)	3.78 (3.77,3.80)	21.8341 (21.4592,22.1729)
			2	228	0.9995	248 (237,272)	249 (236,285)	3.69 (3.68,3.71)	18.9036 (18.5529,19.2308)
			3	208	0.9996	224 (215,245)	222 (213,248)	3.50 (3.49,3.51)	16.0256 (15.7978,16.2866)
		day 135	1	247	0.9994	263 (255,282)	302 (269,385)	2.53 (2.51,2.54)	4.5767 (4.5188,4.6382)
			2	214	0.9993	224 (219,238)	226 (218,248)	2.78 (2.76,2.80)	6.4061 (6.2933,6.5189)
			3	267	0.9994	290 (279,311)	284 (274,306)	2.47 (2.45,2.48)	4.5025 (4.4444,4.5620)
	C1	day 0	1	244	0.9988	305 (281,344)	293 (270,334)	1.66 (1.64,1.68)	2.2707 (2.2457,2.2967)
			2	224	0.9982	277 (255,312)	262 (244,298)	2.00 (1.98,2.02)	2.9586 (2.9095,3.0093)
			3	254	0.9984	314 (291,351)	300 (279,340)	1.94 (1.92,1.96)	2.8050 (2.7647,2.8458)
		day 45	1	156	0.9996	173 (163,198)	177 (163,218)	1.90 (1.89,1.92)	2.4894 (2.4606,2.5189)
			2	187	0.9995	210 (197,237)	222 (200,279)	2.23 (2.22,2.25)	2.9155 (2.8752,2.9568)
			3	138	0.9996	151 (143,172)	162 (145,219)	1.97 (1.95,1.99)	2.5967 (2.5608,2.6344)
		day 90	1	406	0.9979	504 (473,551)	507 (468,572)	2.22 (2.20,2.24)	3.9510 (3.9108,3.9936)
			2	392	0.9983	491 (459,538)	470 (439,520)	2.13 (2.11,2.14)	3.7509 (3.7161,3.7879)
			3	365	0.9967	468 (435,517)	450 (417,505)	2.25 (2.23,2.27)	3.8373 (3.7807,3.8956)
		day 135	1	337	0.9976	442 (407,494)	444 (402,516)	2.22 (2.20,2.24)	3.1486 (3.1017,3.1980)
			2	376	0.9981	480 (447,530)	475 (436,539)	2.04 (2.02,2.06)	2.5530 (2.5221,2.5840)
			3	388	0.9982	471 (443,514)	475 (440,536)	2.42 (2.40,2.43)	3.3201 (3.2723,3.3704)
	C2	day 0	1	321	0.9983	380 (358,414)	366 (346,401)	2.45 (2.44,2.47)	5.3562 (5.2826,5.4318)
			2	328	0.9989	375 (357,404)	366 (349,399)	2.38 (2.36,2.39)	5.1020 (5.0454,5.1626)
			3	301	0.9979	377 (350,419)	370 (341,422)	2.46 (2.44,2.48)	5.2438 (5.1653,5.3277)
		day 45	1	242	0.9984	297 (275,333)	286 (265,325)	1.34 (1.32,1.36)	1.6941 (1.6759,1.7126)
			2	268	0.9990	314 (295,345)	303 (286,336)	1.48 (1.46,1.50)	1.8041 (1.7876,1.8208)
			3	249	0.9978	316 (290,357)	299 (276,341)	1.69 (1.67,1.72)	2.0300 (2.0016,2.0593)
		day 90	1	276	0.9987	336 (313,374)	349 (316,411)	2.64 (2.63,2.66)	6.5920 (6.4977,6.6890)
			2	279	0.9981	380 (344,435)	385 (340,464)	2.60 (2.58,2.62)	6.2893 (6.1958,6.3816)
			3	233	0.9983	279 (260,312)	272 (253,308)	2.56 (2.54,2.58)	6.7024 (6.6050,6.7981)
		day 135	1	298	0.9983	399 (363,453)	376 (343,432)	2.83 (2.81,2.84)	9.4967 (9.3897,9.6061)
			2	263	0.9984	324 (301,363)	319 (293,365)	2.75 (2.73,2.76)	8.2440 (8.1235,8.3682)
			3	250	0.9974	341 (307,393)	341 (301,412)	2.74 (2.72,2.75)	8.3264 (8.2034,8.4531)

Bacteria	NC	day 0	1	Not detectable					
			2						
			3						
		day 45	1	652	0.9967	823 (777,886)	841 (779,932)	4.31 (4.30,4.33)	31.5457 (30.9598,32.1543)
			2	610	0.9952	768 (725,827)	791 (731,881)	4.37 (4.35,4.39)	33.8983 (33.2226,34.7222)
			3	647	0.9958	818 (772,880)	838 (776,930)	4.27 (4.26,4.29)	28.8184 (28.1690,29.4118)
		day 90	1	990	0.9950	1222 (1169,1290)	1201 (1142,1283)	4.99 (4.98,5.01)	61.3497 (60.2410,62.5000)
			2	945	0.9929	1202 (1144,1276)	1202 (1131,1301)	5.09 (5.07,5.11)	72.9927 (71.4286,74.6269)
			3	959	0.9952	1176 (1126,1242)	1165 (1106,1247)	4.96 (4.95,4.98)	57.8035 (56.4972,59.1716)
		day 135	1	1154	0.9945	1363 (1316,1423)	1386 (1322,1473)	5.34 (5.32,5.35)	94.3396 (92.5926,96.1538)
			2	1081	0.9918	1348 (1291,1421)	1358 (1284,1459)	5.32 (5.30,5.34)	86.2069 (84.0336,88.4956)
			3	1162	0.9947	1363 (1318,1422)	1387 (1325,1474)	5.34 (5.32,5.35)	83.3333 (81.3008,85.4701)
	C1	day 0	1	1521	0.9903	1890 (1824,1970)	1920 (1832,2033)	4.36 (4.34,4.39)	14.6628 (14.3472,14.9925)
			2	1552	0.9899	1949 (1880,2033)	1945 (1860,2054)	4.44 (4.42,4.46)	16.4204 (16.0772,16.7785)
			3	1462	0.9882	1859 (1789,1944)	1853 (1768,1963)	4.39 (4.36,4.41)	15.4560 (15.0830,15.8228)
		day 45	1	1830	0.9900	2240 (2170,2324)	2213 (2132,2316)	5.46 (5.44,5.48)	49.2611 (47.8469,50.7614)
			2	2105	0.9976	2174 (2155,2199)	2115 (2110,2125)	5.41 (5.39,5.43)	48.0769 (46.9484,49.2611)
			3	1804	0.9846	2286 (2208,2379)	2270 (2176,2387)	5.40 (5.38,5.43)	45.4545 (44.0529,46.9484)
		day 90	1	2265	0.9840	2926 (2831,3037)	2942 (2820,3091)	5.74 (5.72,5.76)	73.5294 (71.4286,75.7576)
			2	2343	0.9848	3040 (2941,3155)	3008 (2890,3151)	5.78 (5.77,5.79)	80.0000 (77.5194,81.9672)
			3	2207	0.9844	2842 (2749,2951)	2786 (2680,2916)	5.75 (5.73,5.77)	74.0741 (71.9424,76.3359)
		day 135	1	2432	0.9852	3111 (3015,3222)	3087 (2972,3227)	5.78 (5.76,5.80)	69.9301 (68.0272,71.9424)
			2	2198	0.9767	2965 (2857,3091)	2952 (2820,3112)	5.88 (5.86,5.90)	77.5194 (74.6269,80.6452)
			3	2649	0.9866	3320 (3227,3428)	3284 (3174,3417)	5.85 (5.84,5.87)	69.4444 (67.5676,71.4286)
	C2	day 0	1	1606	0.9925	1918 (1860,1989)	1917 (1845,2009)	4.89 (4.87,4.91)	30.3951 (29.6736,31.0559)
			2	1535	0.9916	1904 (1838,1986)	1907 (1823,2015)	4.84 (4.82,4.86)	29.3255 (28.6533,29.9401)
			3	1587	0.9929	1953 (1887,2033)	1947 (1867,2051)	4.76 (4.74,4.78)	27.6243 (27.1003,28.1690)
		day 45	1	1770	0.9895	2328 (2238,2436)	2305 (2197,2440)	5.41 (5.39,5.43)	71.9424 (70.4225,73.5294)
			2	1640	0.9906	2101 (2022,2197)	2111 (2011,2239)	5.33 (5.31,5.35)	68.9655 (67.5676,70.4225)
			3	1635	0.9917	2009 (1942,2091)	1986 (1908,2086)	5.39 (5.37,5.40)	75.1880 (74.0741,76.9231)
		day 90	1	2157	0.9887	2689 (2606,2786)	2696 (2592,2826)	5.88 (5.86,5.90)	109.8901 (107.5269,112.3596)
			2	2340	0.9898	2849 (2771,2942)	2855 (2756,2976)	5.95 (5.93,5.96)	114.9425 (112.3596,117.6471)
			3	2111	0.9884	2582 (2507,2672)	2570 (2478,2684)	5.90 (5.89,5.92)	109.8901 (107.5269,113.6364)
		day 135	1	1984	0.9859	2548 (2460,2652)	2551 (2442,2687)	5.17 (5.15,5.20)	15.6250 (15.1515,16.1290)
			2	2145	0.9890	2668 (2587,2764)	2649 (2552,2770)	5.20 (5.18,5.23)	15.8730 (15.4560,16.3399)
			3	1829	0.9855	2390 (2302,2495)	2359 (2256,2489)	4.58 (4.55,4.61)	7.9745 (7.7760,8.1900)

Table S3 Chemical composition of major nutrients in the fruiting bodies harvested from the NC, C1, and C2 substrata, respectively. All the examined nutrients showed no significant difference in their contents among the three substrata, as tested by one-way ANOVA (all P>0.05).

Content (mg g^{-1} dry-weight)	NC	C1	C2
Total organic C	407.92±34.03	423.72±19.26	421.27±33.41
Total N	36.23±0.89	38.97±2.35	37.38±2.91
Total P	4.44±0.29	4.75±0.23	4.31±0.18
Total K	7.63±0.34	7.53±0.44	7.35±0.39
Total carbohydrates	665.06±31.74	621.23±18.61	625.02±30.33
Total lipids	108.61±21.43	129.06±13.16	115.36±8.14
Total proteins	29.62±3.31	25.39±2.96	26.52±1.89
Free amino acids	12.49±1.10	13.08±0.49	11.91±1.85

Table S4 Functional prediction of fungal OTUs by FUNGuild, and bacterial OTUs by FAPROTAX. The table is of big size and is therefore provided as multiple working-sheets in an individual Excel file available online: TableS4.xlsx.

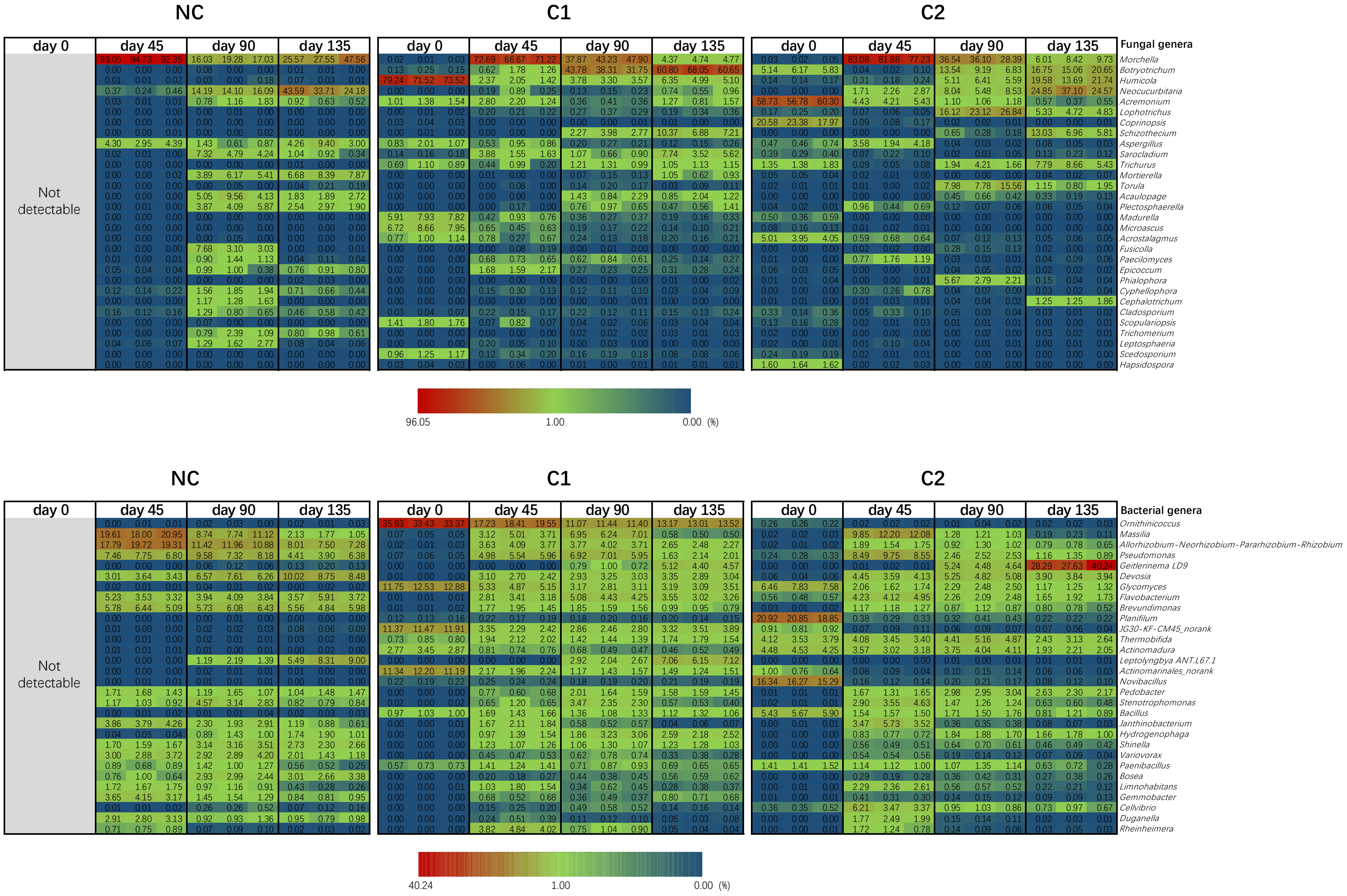

Figure S2 Heatmap of fungal and bacterial genera, showing the top 30 prominent genera in the samples.

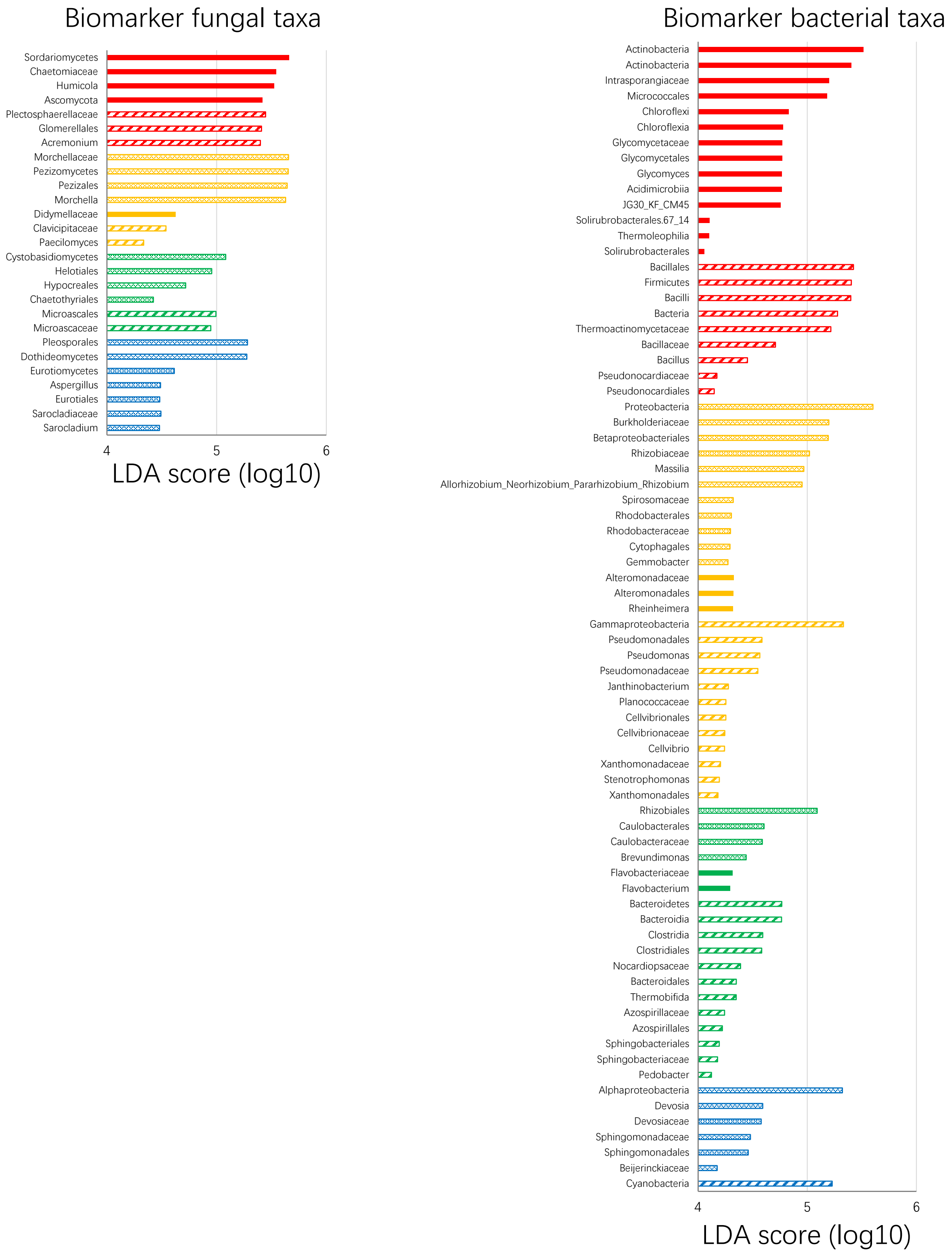

Figure S3 Biomarker taxa of the substrata at different time-points, determined by LEfSe. The bar-charts show the taxa with a LDA score (log10)>4.0 and P-value<0.05. Samples of NC, C1, and C2 are indicated by empty boxes filled with dots, solid bars, and empty boxes filled with diagonal stripes, respectively. Samples at day 0, 45, 90, and 135 are colored in red, orange, green, and blue, respectively.

Ecological guild of fungi	NC day 0	NC day 45	NC day 90	NC day 135	C1 day 0	C1 day 45	C1 day 90	C1 day 135	C2 day 0	C2 day 45	C2 day 90	C2 day 135
Undefined Saprotroph	Not detectable	4.48 3.05 4.56	26.51 14.93 15.88	6.03 11.29 3.80	3.11 5.27 4.08	7.63 7.21 6.44	48.04 42.06 35.78	71.12 75.30 71.75	9.79 10.77 10.86	4.90 4.47 6.24	30.64 33.16 34.45	24.02 21.80 27.89
Undefined Saprotroph-Wood Saprotroph		0.01 0.01 0.02	0.04 0.07 0.24	0.08 0.03 0.03	79.32 71.61 73.60	2.62 2.36 1.65	3.90 3.39 3.72	6.49 5.16 5.34	0.16 0.16 0.19	0.33 0.19 0.25	5.19 6.51 5.68	19.82 13.86 21.94
Wood Saprotroph		0.00 0.00 0.00	0.06 0.00 0.08	0.78 1.50 0.81	0.69 1.10 0.90	0.49 1.14 0.23	1.27 1.36 1.05	1.08 1.17 1.21	1.65 1.52 1.96	0.10 0.10 0.11	1.97 4.28 1.68	7.88 8.76 5.48
Dung Saprotroph		0.00 0.00 0.03	0.00 0.00 0.03	0.04 0.17 0.05	0.01 0.02 0.01	0.00 0.00 0.00	2.36 4.10 2.92	10.60 7.13 7.56	0.01 0.01 0.02	0.00 0.00 0.00	0.83 0.81 0.45	13.38 7.19 6.22
Dung Saprotroph-Plant Saprotroph-Wood Saprotroph		0.01 0.00 0.00	0.00 0.00 0.00	0.01 0.01 0.01	0.03 0.04 0.03	0.00 0.00 0.00	0.00 0.00 0.01	0.01 0.00 0.00	20.67 23.47 18.03	0.10 0.08 0.17	0.02 0.02 0.01	0.00 0.00 0.01
Ectomycorrhizal-Undefined Saprotroph-Wood Saprotroph		94.48 96.05 93.97	20.43 23.88 22.94	27.47 29.38 50.47	0.02 0.01 0.03	80.30 76.84 82.63	38.99 44.28 49.96	4.46 4.91 5.00	0.03 0.02 0.05	85.45 85.79 80.76	37.15 36.66 28.82	6.09 8.52 9.82
Endophyte-Undefined Saprotroph		0.00 0.00 0.00	0.00 0.06 0.00	0.00 0.00 0.00	0.77 1.00 1.14	0.86 0.31 0.77	0.25 0.13 0.19	0.21 0.17 0.22	5.03 3.97 4.07	0.61 0.71 0.66	0.07 0.12 0.13	0.05 0.06 0.05
Endophyte-Litter Saprotroph-Soil Saprotroph-Undefined Saprotroph		0.00 0.00 0.02	4.96 7.65 7.29	7.18 8.95 8.35	0.00 0.00 0.00	0.01 0.00 0.00	0.08 0.15 0.14	1.07 0.64 0.97	0.05 0.05 0.04	0.02 0.01 0.00	0.00 0.00 0.00	0.04 0.02 0.07
Endophyte		0.00 0.00 0.02	1.31 3.19 1.75	0.87 1.06 0.65	0.00 0.00 0.00	0.00 0.00 0.05	0.02 0.02 0.02	0.03 0.01 0.03	0.03 0.00 0.03	0.01 0.00 0.02	0.01 0.03 0.00	0.03 0.02 0.01
Endophyte-Plant Pathogen-Wood Saprotroph		0.00 0.00 0.01	0.46 0.48 0.57	0.37 0.55 0.35	0.02 0.02 0.01	0.10 0.13 0.00	0.19 0.24 0.24	0.07 0.12 0.22	0.34 0.17 0.19	0.01 0.01 0.03	8.12 7.90 15.81	1.18 0.82 1.98
Plant Pathogen-Wood Saprotroph		0.38 0.25 0.47	18.10 17.46 21.73	46.87 36.12 25.74	0.00 0.00 0.00	0.21 1.02 0.29	0.14 0.15 0.24	0.87 0.66 1.19	0.01 0.01 0.01	1.76 2.36 3.00	8.17 5.56 8.66	25.15 37.56 24.80
Plant Pathogen		0.09 0.08 0.14	7.25 8.22 12.32	3.26 3.46 2.21	0.17 0.24 0.32	0.25 0.88 0.27	0.85 1.08 0.72	0.54 0.64 1.54	0.35 0.33 0.30	1.06 0.59 0.79	0.14 0.08 0.07	0.07 0.05 0.05
Animal Pathogen		0.01 0.00 0.02	6.64 11.94 5.56	2.13 2.11 3.10	5.91 7.94 7.83	0.64 1.08 0.88	1.88 1.18 2.81	1.07 2.28 1.63	0.51 0.37 0.60	0.00 0.00 0.00	0.46 0.67 0.43	0.34 0.19 0.13
Animal Pathogen-Dung Saprotroph-Endophyte-Epiphyte-Plant Saprotroph-Wood Saprotroph		0.00 0.00 0.00	0.06 0.01 0.00	0.03 0.05 0.02	0.56 0.67 0.57	0.24 1.29 0.55	0.10 0.10 0.08	0.05 0.07 0.17	1.14 1.06 1.38	0.12 0.14 0.16	0.06 0.06 0.05	0.06 0.02 0.05
Animal Pathogen-Endophyte-Ericoid Mycorrhizal-Plant Pathogen-Wood Saprotroph		0.00 0.00 0.00	0.00 0.00 0.00	0.03 0.03 0.00	0.00 0.00 0.00	0.00 0.00 0.00	0.00 0.00 0.00	0.01 0.00 0.02	0.01 0.01 0.04	0.00 0.00 0.01	5.76 2.83 2.25	0.15 0.04 0.04
Animal Pathogen-Endophyte-Fungal Parasite-Plant Pathogen-Wood Saprotroph		0.03 0.01 0.01	0.99 1.43 2.46	0.98 0.67 0.55	1.02 1.39 1.55	3.09 2.54 1.44	0.37 0.42 0.38	1.30 0.84 1.65	58.99 57.05 60.50	4.55 4.41 5.67	1.12 1.07 1.20	0.57 0.37 0.55
Animal Pathogen-Endophyte-Lichen Parasite-Plant Pathogen-Wood Saprotroph		0.16 0.13 0.16	1.65 0.99 0.87	0.50 0.62 0.44	0.03 0.04 0.07	0.24 0.17 0.19	0.22 0.13 0.12	0.15 0.13 0.26	0.33 0.14 0.36	0.05 0.35 0.10	0.05 0.03 0.04	0.03 0.01 0.02
Animal Pathogen-Endophyte-Plant Pathogen-Undefined Saprotroph		0.00 0.00 0.00	0.00 0.00 0.00	0.00 0.00 0.00	6.73 8.67 7.96	0.71 0.52 0.73	0.20 0.18 0.23	0.14 0.11 0.22	0.08 0.16 0.14	0.01 0.03 0.01	0.00 0.00 0.00	0.00 0.00 0.00
Animal Pathogen-Plant Pathogen-Undefined Saprotroph		0.05 0.04 0.04	1.50 1.24 0.73	0.87 1.06 0.85	0.02 0.02 0.01	1.94 1.84 2.71	0.28 0.25 0.28	0.32 0.29 0.27	0.09 0.04 0.06	0.04 0.03 0.06	0.04 0.05 0.02	0.02 0.02 0.02
Animal Pathogen-Undefined Saprotroph		0.13 0.15 0.23	2.74 2.43 2.77	0.82 0.72 0.48	0.00 0.01 0.00	0.17 0.39 0.26	0.13 0.12 0.11	0.03 0.05 0.09	0.00 0.02 0.01	0.31 0.28 0.82	0.04 0.08 0.10	0.03 0.02 0.04

Colour scale: 96.05 — 1.00 — 0.00 (%)

Figure S4 Heatmap of ecological guilds in the fungal communities, showing only the top 30 prominent guilds. Guild belonging to a trophic mode of saprotroph or facultative saprotroph-symbiotroph were colored in blue. Symbiotrophic guilds in green. Guilds with mainly pathotrophic nutrition in red.

NC

Ecological group of bacteria	day 0	day 45			day 90			day 135		
cellulolysis		75.22	90.43	75.85	104.34	101.57	85.72	114.79	133.75	84.55
xylanolysis		0.26	0.52	0.28	1.14	0.80	0.43	1.72	1.61	0.20
ligninolysis		41.62	54.87	48.60	18.37	19.12	18.79	21.46	14.06	19.05
chitinolysis		9.56	15.97	9.27	8.71	8.36	8.64	11.80	27.71	16.27
fermentation		156.65	135.00	147.48	54.16	57.75	94.14	33.04	40.97	33.14
nitrogen_fixation		78.32	80.64	59.55	71.20	79.66	73.63	57.93	47.80	48.23
nitrate_ammonification		0.26	0.26	0.00	0.00	0.00	0.43	0.00	0.40	0.00
nitrite_ammonification		0.26	0.26	0.00	0.00	0.00	0.43	0.00	0.40	0.00
anammox		0.00	0.00	0.00	0.00	0.00	0.00	0.00	0.00	0.00
nitrification		0.00	1.29	0.28	0.00	0.00	0.00	0.64	0.20	0.00
aerobic_ammonia_oxidation	Not detectable	0.00	1.29	0.28	0.00	0.00	0.00	0.64	0.20	0.00
aerobic_nitrite_oxidation		0.00	0.00	0.00	0.00	0.00	0.00	0.00	0.00	0.00
denitrification		45.50	60.03	54.50	21.40	21.31	21.16	18.67	12.65	19.45
nitrate_denitrification		4.14	5.41	6.74	3.60	3.39	4.32	2.57	3.21	3.77
nitrite_denitrification		4.14	5.41	6.74	3.60	3.39	4.32	2.57	3.21	3.77
nitrous_oxide_denitrification		4.14	5.41	6.74	3.60	3.39	4.32	2.57	3.21	3.77
nitrate_reduction		561.20	575.28	532.33	463.75	379.58	406.37	183.24	173.51	190.33
nitrate_respiration		403.52	434.10	383.73	413.76	329.80	336.62	130.46	126.52	133.37
nitrite_respiration		4.39	5.67	6.74	3.60	3.58	4.75	2.57	3.61	3.77
nonphotosynthetic_cyanobacteria		0.00	0.00	0.00	0.00	0.00	0.00	0.00	0.00	0.00
photosynthetic_cyanobacteria		0.00	0.00	0.84	79.34	149.96	100.62	392.44	546.85	577.75
intracellular_parasites		5.69	7.99	10.39	13.07	14.34	15.98	9.66	6.23	8.14

C1

Ecological group of bacteria	day 0			day 45			day 90			day 135		
cellulolysis	85.11	100.41	94.28	219.89	217.40	216.01	200.41	230.59	220.90	187.12	207.23	179.05
xylanolysis	110.44	131.54	127.82	170.92	165.32	170.68	88.12	89.87	88.21	111.23	117.10	102.99
ligninolysis	0.00	0.00	0.00	59.26	55.48	58.98	99.32	124.55	107.49	37.20	49.94	43.81
chitinolysis	1.35	0.70	1.06	5.88	8.38	3.93	13.68	13.12	8.90	3.89	5.55	5.50
fermentation	54.71	54.93	66.03	110.68	127.50	92.05	47.26	45.47	60.42	40.35	57.02	55.00
nitrogen_fixation	4.73	5.25	7.06	15.92	8.15	12.26	8.35	9.71	8.15	12.03	17.60	15.55
nitrate_ammonification	1.01	1.40	0.71	0.98	1.36	2.31	0.53	0.54	0.93	0.37	0.57	0.57
nitrite_ammonification	1.01	1.40	0.71	1.22	2.04	2.78	0.89	0.54	1.11	0.37	0.77	0.57
anammox	0.00	0.00	0.00	0.00	0.00	0.00	0.00	0.00	0.00	0.00	0.00	0.00
nitrification	6.08	5.95	4.24	1.71	0.23	1.85	0.18	1.08	0.56	0.19	0.57	0.19
aerobic_ammonia_oxidation	3.72	4.20	3.53	1.71	0.23	1.85	0.18	0.90	0.56	0.19	0.57	0.19
aerobic_nitrite_oxidation	2.36	1.75	0.71	0.00	0.00	0.00	0.00	0.18	0.00	0.00	0.00	0.00
denitrification	1.01	0.70	0.00	68.07	62.28	68.69	99.50	125.09	110.27	40.16	50.71	46.09
nitrate_denitrification	1.01	0.70	0.00	10.04	7.70	11.33	2.31	4.31	3.15	3.52	2.49	2.47
nitrite_denitrification	1.01	0.70	0.00	10.04	7.70	11.33	2.31	4.31	3.15	3.52	2.49	2.47
nitrous_oxide_denitrification	1.01	0.70	0.00	10.04	7.70	11.33	2.31	4.31	3.15	3.52	2.49	2.47
nitrate_reduction	15.87	19.24	13.77	178.26	211.51	179.47	272.01	232.21	225.54	121.60	121.70	114.94
nitrate_respiration	8.11	7.35	5.30	129.53	154.67	127.90	235.77	190.51	180.13	94.02	85.34	79.28
nitrite_respiration	2.70	3.50	2.12	11.26	12.91	15.26	3.73	5.21	4.63	5.18	3.64	4.55
nonphotosynthetic_cyanobacteria	1.35	2.45	2.12	0.24	1.36	0.69	0.71	0.54	0.56	0.74	0.77	0.19
photosynthetic_cyanobacteria	0.00	0.00	0.35	0.00	0.23	0.23	215.51	179.55	205.71	809.18	729.99	782.01
intracellular_parasites	0.34	1.40	2.47	2.20	1.36	1.16	8.17	5.57	5.56	9.07	9.18	9.67

C2

Ecological group of bacteria	day 0			day 45			day 90			day 135		
cellulolysis	486.75	430.50	444.74	335.65	293.30	285.15	340.43	382.45	374.93	169.75	205.95	136.95
xylanolysis	539.39	469.08	474.30	259.11	212.92	199.25	260.45	309.61	298.72	111.57	145.11	106.57
ligninolysis	0.00	0.38	0.00	69.32	70.03	72.63	37.36	46.55	43.58	16.72	18.00	10.17
chitinolysis	5.93	4.97	5.47	19.53	18.64	21.34	9.43	6.94	12.05	4.78	8.71	6.78
fermentation	229.10	231.87	230.58	123.60	138.37	114.60	210.21	221.62	219.73	95.83	88.84	52.22
nitrogen_fixation	1.11	1.15	0.73	64.24	67.96	50.22	217.28	196.79	183.57	126.61	77.70	64.77
nitrate_ammonification	1.11	1.15	1.46	12.69	24.29	12.02	1.81	1.10	1.48	0.70	0.71	0.38
nitrite_ammonification	1.11	1.15	1.46	12.69	24.29	12.02	1.81	1.10	1.67	0.70	0.71	0.50
anammox	0.00	0.00	0.00	0.00	0.00	0.00	0.00	0.00	0.00	0.00	0.00	0.00
nitrification	0.00	0.38	0.73	0.00	0.00	0.00	0.36	0.00	0.56	0.42	0.43	0.63
aerobic_ammonia_oxidation	0.00	0.38	0.73	0.00	0.00	0.00	0.36	0.00	0.56	0.42	0.43	0.63
aerobic_nitrite_oxidation	0.00	0.00	0.00	0.00	0.00	0.00	0.00	0.00	0.00	0.00	0.00	0.00
denitrification	25.95	21.01	21.53	72.44	75.68	76.22	42.62	47.46	44.50	17.00	16.57	10.54
nitrate_denitrification	25.95	20.63	21.53	4.30	6.78	5.02	10.52	4.75	5.19	1.41	0.86	1.26
nitrite_denitrification	25.95	20.63	21.53	4.30	6.78	5.02	10.52	4.75	5.19	1.41	0.86	1.26
nitrous_oxide_denitrification	25.95	20.63	21.53	4.30	6.78	5.02	10.52	4.75	5.19	1.41	0.86	1.26
nitrate_reduction	127.15	121.85	126.60	342.09	390.63	416.43	229.43	201.54	202.67	108.20	109.40	66.28
nitrate_respiration	78.96	76.02	83.91	288.01	325.87	362.27	169.22	155.53	149.82	54.66	52.99	38.41
nitrite_respiration	29.29	24.07	25.90	20.11	34.07	19.55	13.78	9.31	10.38	2.81	1.86	2.38
nonphotosynthetic_cyanobacteria	0.74	0.00	0.00	0.20	0.00	0.00	0.54	0.37	0.19	0.00	0.00	0.25
photosynthetic_cyanobacteria	0.00	0.38	0.00	0.78	1.32	0.54	309.05	270.72	281.11	1313.15	1301.42	1630.70
intracellular_parasites	1.48	0.76	0.00	3.71	4.52	5.02	14.87	10.95	12.24	12.08	9.57	10.67

1630.70 16.31 0.00 $(\times 10^{-4})$

Figure S5 Heatmap of functional groups in the bacterial communities predicted by FAXPROTAX, showing the groups related intracellular parasitism and N-metabolism.

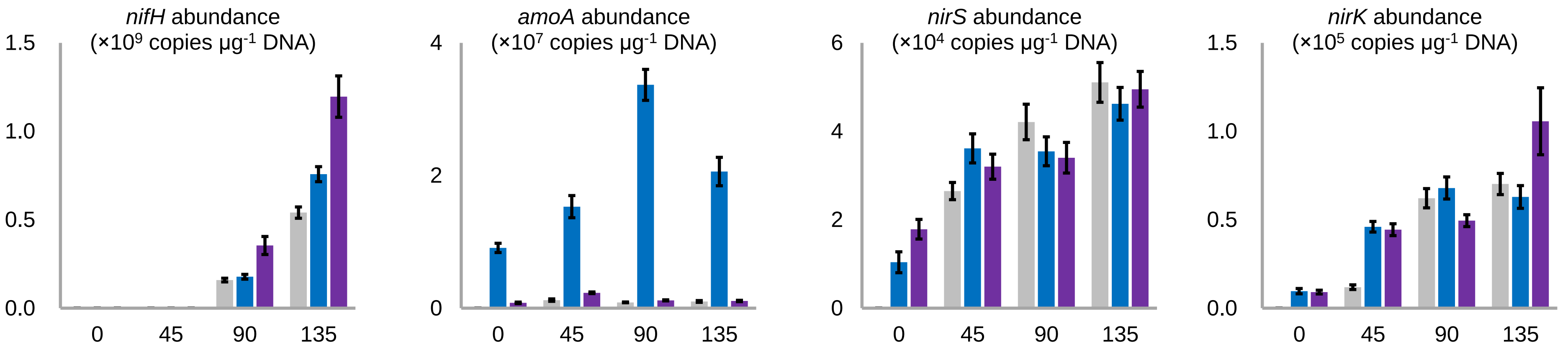

Figure S6 qPCR estimation of the abundances of marker genes involved in N-fixation, nitrification, and denitrification. The horizontal axis in all charts means days after morel sowing.

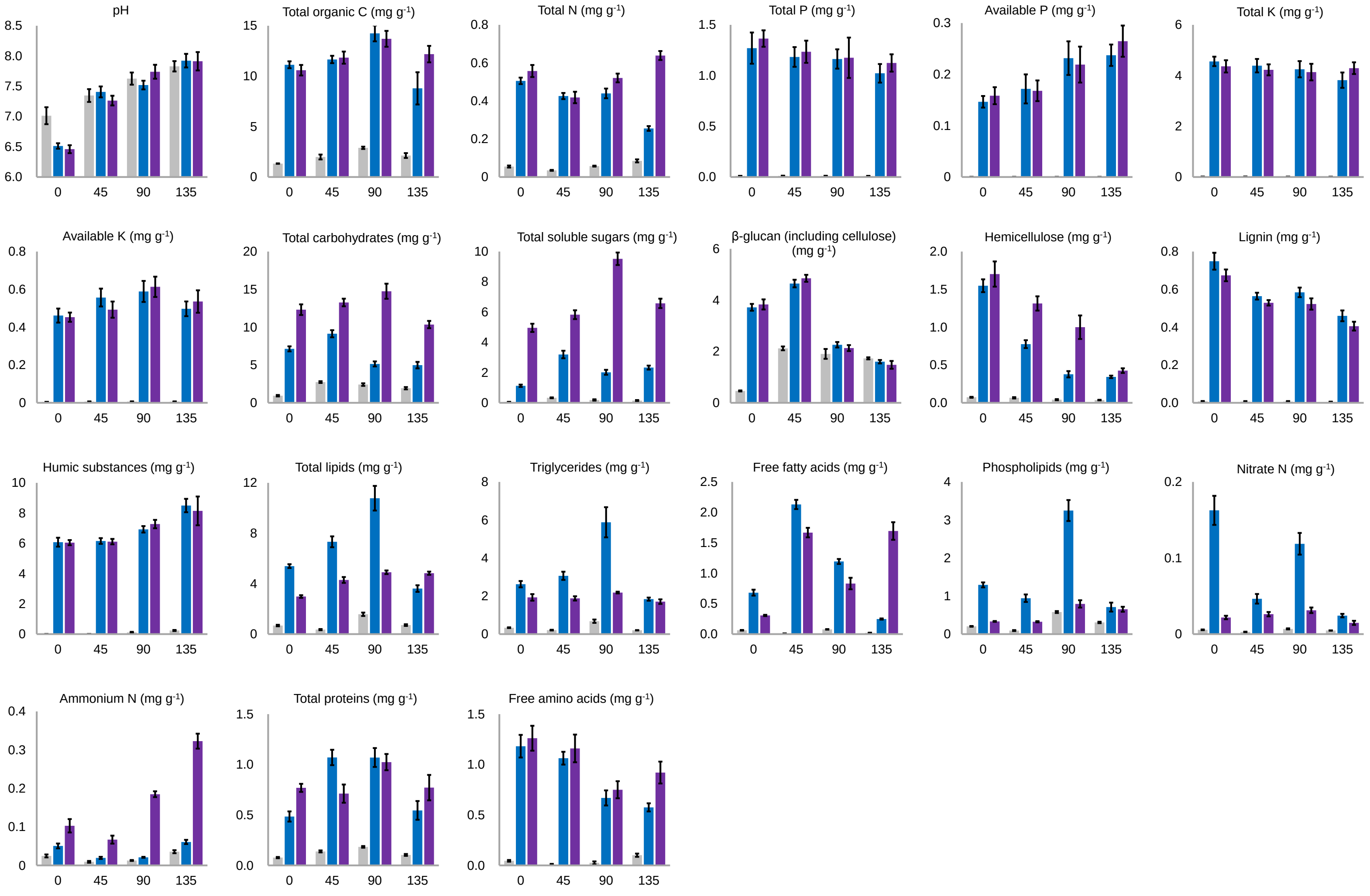

Figure S7 Contents of the major nutritional substances in the substrata. NC, C1, and C2 were colored in grey, blue, and purple, respectively. All the presented values are the mean of three biological replicates with standard deviation bars. The horizontal axis in the charts means days after morel sowing.

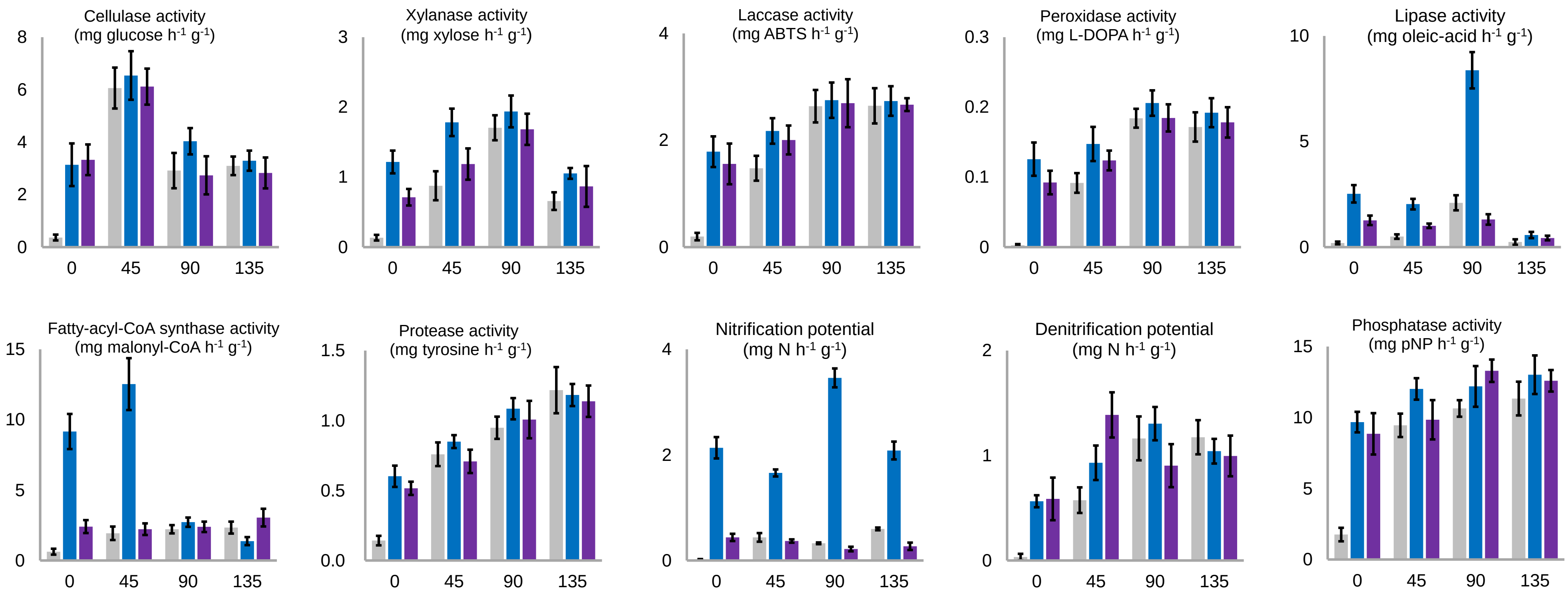

Figure S8 Activity levels of key enzymes involved in the metabolism and transformation of nutritional substances. The activity level was shown as the catalytic ability to transform one milligram of the assay-substrate per hour by one gram (dry weight) of the substratum. NC, C1, and C2 were colored in grey, blue, and purple, respectively. All the presented values are the mean of three biological replicates with standard deviation bars. The horizontal axis in the charts means days after morel sowing.
